# Supplementary material for: Cross‐tissue comparison of epigenetic aging clocks in humans
Source: Aging Cell. 2025 Jan 9;24(4):e14451. doi: 10.1111/acel.14451 (PMC11984668; doi:10.1111/acel.14451)
Supplement: Supplementary file 1 — Appendix S1. [file ACEL-24-e14451-s001.pdf]

Section S1. Supplementary Methods and Materials

S1.1. EPIC v1 and EPIC v2 Illumina Methylation Arrays

Most epigenetic clocks have been constructed using the probes present on either the 27k, 450k, or EPIC v1 Illumina chips. However, due to the recent development and release of the EPIC v2 chip, some probes used to generate epigenetic clocks are either missing or duplicated in the EPIC v2 chip. All samples in our study were assayed using the EPIC v2 array, therefore, comparisons between the two arrays were not made in the current study.

Although we did not use any EPIC v1 arrays in the current project, we present the following information to promote accurate comparability between epigenetic clocks generated using EPIC v1 and EPIC v2 arrays. To compare epigenetic clocks calculated with EPIC v1 and EPIC v2 arrays, two main concerns need to be addressed:

1. How should probes in epigenetic clock algorithms that contain duplicate probes on the EPIC v2 chip be addressed?
2. How should probes in epigenetic clock algorithms that are missing on the EPIC v2 chip be addressed?

S1.1.1 – Duplicated Probes

In order to remove duplicate probes in the EPIC v2 chip, we used the R package *ENmix* (using v1.36.08 or newer). This package has a function called *rm.cgsuffix*. This function both removes the suffix on EPIC v2 probe names (i.e., removes "_BC11" from "cg25324105_BC11") and collapses duplicate probes by taking their averages.

S1.1.2 – Missing Probes

When EPIC v2 chips are used, each epigenetic clock has a specific number of probes that are missing from the original algorithm. For example, the DunedinPACE algorithm is missing 29 of its original 173 probes used. Currently, the solution to these missing probes is to impute them. The word imputation is used loosely here, because if a probe is missing in all samples, the process of estimating the probe values is technically considered "extrapolation". Regardless, the values of these missing probes are imputed/extrapolated by being set equal to the mean of a "golden standard" (GS) dataset, with each dataset differing by the clock being used. This means that all samples receive the exact same value (the mean of the GS dataset) for missing probes, thereby decreasing variation in the estimated values of the epigenetic clock.

S1.2. "Golden Standard" Datasets for Imputation

In our current work, we have opted to use the imputation/extrapolation method mentioned above, where the probe values are replaced with the mean of the GS dataset probes. However, because there are different GS datasets used for each clock, and each of these datasets is based on a single tissue type, we generated custom GS datasets stratified by tissue and age. This resulted in a total of 8 GS datasets: adult buccal, children buccal, adult saliva, children saliva, adult whole blood, children whole blood, adult PBMCs and

children PBMCs. Each GS dataset was constructed using 2 publicly available datasets, one using the EPIC v1 array and another using the 450k array. All datasets with available IDAT files were preprocessed using the same procedures described in the manuscript (see *Methods*). By necessity, all datasets with raw intensity files available excluded the bisulfite conversion and bead count filtering steps. All publicly available datasets were BMIQ normalized before the generation of GS datasets. (See **Table S2** for dataset details).

S1.3. Estimations of Cellular Composition

Because cell types have unique DNA methylation signatures, the cellular composition of a tissue sample can be a confounding factor when estimating epigenetic clocks. We performed estimations of cellular composition for each tissue in the current study using two distinct methods.

S1.3.1 – The Houseman-Blood Method

The first method, which we refer to as the "Houseman-Blood" method was used for all blood-based tissues (dry blood spots (DBS), buffy coat, and peripheral blood mononuclear cells (PBMCs)). The Houseman-Blood method, which has been widely used for estimating immune cell compositions and has been detailed previously (Houseman et al. 2012), estimates cellular proportions of CD8T, CD4T, natural killer cells, B-cells, monocytes and granulocytes from DNA methylation data. These estimates are then used when controlling for cellular compositions of blood-based tissues.

S1.3.2 – The Houseman-ReferenceFree Method

The second method, which we refer to as the "Houseman-ReferenceFree" method was used for buccal and saliva tissue. The Houseman-ReferenceFree method can be used for any type of tissue (including non-blood tissue) and will provide estimates for cellular proportions based on DNA methylation data and a user-defined input that specifies the number of cell types to estimate (Houseman et al. 2016). In addition to providing cellular proportions, the Houseman-ReferenceFree method also provides a protocol for estimating the "best-fitting" number of cell subtypes in a provided sample. Briefly, deviance statistics are computed for each possible number of cell types present (2 cell types, 3 cell types, …). These deviance statistics are computed for a random selection of the study sample a defined number of times (we used 1000 bootstrapped samples). Then, summary statistics for each possible number of cell types present are compared and the number of cell types with the minimum deviance summary statistic are chosen.

We performed these calculations with our buccal and saliva samples separately. We extracted the 10,000 probes with the highest variation in buccal and saliva separately and subsequently used these probes in cellular composition estimates. After computation, we determined that buccal tissue contained 3 cellular subtypes and saliva contained 5 (see **Figure S1**).

Section S2. Supplementary Results and Figures

S2.1. Supplementary Tables

Epigenetic Clock	Total CpG Probes	Number of Probes Present in EPIC v2	Number of Probes Absent in EPIC v2	Proportion Missing
Horvath	353	340	13	3.68%
Hannum	71	64	7	9.86%
PhenoAge	513	495	18	3.51%
GrimAge2	1030	845	185	17.96%
DunedinPACE	173	144	29	16.76%
PedBE	94	93	1	1.06%
Skin and Blood	391	374	17	4.35%

Table S1: An overview of the number of CpG probes included in the EPIC v2 array for each clock used in the present analysis.

GEO Accession Number	Tissue	Sample Number	Sample Inclusion Criteria	Age	Array	Raw IDAT Used	Intensity Matrix Used	Sample Pval Filter	Sample Bisulfite Converstion Filter	Probe Bead Count Filter	Probe Pval Filter	Normalization
GSE50586	Buccal	10	Only controls included	Adult	450k	No	Yes	Yes	No	No	Yes	BMIQ
GSE166844	Buccal	28	Only buccal samples	Adult	EPIC v1	No	Yes	Yes	No	No	Yes	BMIQ
GSE50759	Buccal	40	Only controls included	Children	450k	No	Yes	Yes	No	No	Yes	BMIQ
GSE147058	Buccal	48	Only twin A included	Children	EPIC v1	Yes	No	Yes	Yes	Yes	Yes	BMIQ
GSE245924	PBMC	16	Only controls included	Adult	EPIC v1	Yes	No	Yes	Yes	Yes	Yes	BMIQ
GSE117929	PBMC	19	Only controls included	Adult	450k	No	Yes	Yes	No	No	Yes	BMIQ
GSE132181	PBMC	50	Only PBMC age 7 included, used first 50 samples	Children	EPIC v1	Yes	No	Yes	Yes	Yes	Yes	BMIQ
GSE40576	PBMC	30	Only first 30 controls included	Children	450k	No	Yes	Yes	No	No	Yes	BMIQ
GSE130153	Saliva	22	Only saliva test samples included	Adult	450k	No	Yes	Yes	No	No	Yes	BMIQ
GSE111165	Saliva	15	Only EPIC saliva samples over 18	Adult	EPIC v1	Yes	No	Yes	Yes	Yes	Yes	BMIQ
GSE147318	Saliva	18	Only whole saliva samples	Children	EPIC v1	Yes	No	Yes	Yes	Yes	Yes	BMIQ
GSE112314	Saliva	22	All samples included	Children	450k	Yes	No	Yes	Yes	Yes	Yes	BMIQ
GSE218186	Whole Blood	55	Only controls included	Adult	EPIC v1	Yes	No	Yes	Yes	Yes	Yes	BMIQ
GSE201287	Whole Blood	40	Only controls included	Adult	450k	Yes	No	Yes	Yes	Yes	Yes	BMIQ
GSE221864	Whole Blood	31	Only controls included	Children	EPIC v1	Yes	No	Yes	Yes	Yes	Yes	BMIQ
GSE174555	Whole Blood	17	Only controls included	Children	450k	Yes	No	Yes	Yes	Yes	Yes	BMIQ

Table S2: A description of the public datasets and preprocessing methods used to construct custom "golden standard" (GS) datasets for imputation of missing CpGs.

	Horvath Pan-Tissue Acceleration							Hannum Acceleration						
	**ICC**	**F**	**df1**	**df2**	**P-value**	**Lower Bound**	**Upper Bound**	**ICC**	**F**	**df1**	**df2**	**P-value**	**Lower Bound**	**Upper Bound**
**ICC1**	0.09	1.5	82	332	0.009	0.01	0.18	-0.05	0.76	82	332	0.930	-0.10	0.02
**ICC2**	0.17	3.1	82	328	<0.001	0.06	0.30	0.11	5.29	82	328	<0.001	0.01	0.25
**ICC3**	0.30	3.1	82	328	<0.001	0.20	0.41	0.46	5.29	82	328	<0.001	0.36	0.57
**ICC1k**	0.32	1.5	82	332	0.009	0.06	0.53	-0.31	0.76	82	332	0.930	-0.82	0.09
**ICC2k**	0.50	3.1	82	328	<0.001	0.23	0.68	0.38	5.29	82	328	<0.001	0.06	0.62
**ICC3k**	0.68	3.1	82	328	<0.001	0.56	0.78	0.81	5.29	82	328	<0.001	0.74	0.87

Table S3: Intraclass correlation coefficients across all tissues for the Horvath-Pan Tissue and Hannum acceleration estimates. ICC1 - One-way random effects, absolute agreement, single rater/measurement, ICC2 - Two-way random effects, absolute agreement, single rater/measurement, ICC3 - Two-way mixed effects, consistency, single rater/measurement, ICC1k - One-way random effects, absolute agreement, multiple raters/measurements, ICC2k - Two-way random effects, absolute agreement, multiple raters/measurements, ICC3k - Two-way mixed effects, consistency, multiple raters/measurements. F – Fisher's F statistic, df – degrees of freedom.

	Skin and Blood Acceleration							PedBE Acceleration						
	**ICC**	**F**	**df1**	**df2**	**P-value**	**Lower Bound**	**Upper Bound**	**ICC**	**F**	**df1**	**df2**	**P-value**	**Lower Bound**	**Upper Bound**
**ICC1**	0.18	2.1	82	332	<0.001	0.09	0.28	0.67	11	82	332	<0.001	0.58	0.75
**ICC2**	0.19	2.3	82	328	<0.001	0.11	0.30	0.68	42	82	328	<0.001	0.34	0.84
**ICC3**	0.21	2.3	82	328	<0.001	0.12	0.32	0.89	42	82	328	<0.001	0.85	0.92
**ICC1k**	0.52	2.1	82	332	<0.001	0.33	0.66	0.91	11	82	332	<0.001	0.87	0.94
**ICC2k**	0.54	2.3	82	328	<0.001	0.37	0.68	0.92	42	82	328	<0.001	0.72	0.96
**ICC3k**	0.57	2.3	82	328	<0.001	0.40	0.70	0.98	42	82	328	<0.001	0.97	0.98

Table S4: Intraclass correlation coefficients across all tissues for the Skin and Blood and PedBE acceleration estimates. ICC1 - One-way random effects, absolute agreement, single rater/measurement, ICC2 - Two-way random effects, absolute agreement, single rater/measurement, ICC3 - Two-way mixed effects, consistency, single rater/measurement, ICC1k - One-way random effects, absolute agreement, multiple raters/measurements, ICC2k - Two-way random effects, absolute agreement, multiple raters/measurements, ICC3k - Two-way mixed effects, consistency, multiple raters/measurements. F – Fisher's F statistic, df – degrees of freedom.

	PhenoAge Acceleration							GrimAge2 Acceleration						
	**ICC**	**F**	**df1**	**df2**	**P-value**	**Lower Bound**	**Upper Bound**	**ICC**	**F**	**df1**	**df2**	**P-value**	**Lower Bound**	**Upper Bound**
**ICC1**	-0.13	0.45	82	332	1.000	-0.16	-0.08	-0.05	0.76	82	332	0.930	-0.10	0.02
**ICC2**	0.05	2.39	82	328	<0.001	0.00	0.12	0.12	7.61	82	328	<0.001	0.01	0.27
**ICC3**	0.22	2.39	82	328	<0.001	0.13	0.33	0.57	7.61	82	328	<0.001	0.47	0.67
**ICC1k**	-1.25	0.45	82	332	1.000	-2.11	-0.56	-0.31	0.76	82	332	0.930	-0.82	0.08
**ICC2k**	0.21	2.39	82	328	<0.001	0.01	0.40	0.40	7.61	82	328	<0.001	0.06	0.64
**ICC3k**	0.58	2.39	82	328	<0.001	0.42	0.71	0.87	7.61	82	328	<0.001	0.82	0.91

Table S5: Intraclass correlation coefficients across all tissues for the PhenoAge and GrimAge2 acceleration estimates. ICC1 - One-way random effects, absolute agreement, single rater/measurement, ICC2 - Two-way random effects, absolute agreement, single rater/measurement, ICC3 - Two-way mixed effects, consistency, single rater/measurement, ICC1k - One-way random effects, absolute agreement, multiple raters/measurements, ICC2k - Two-way random effects, absolute agreement, multiple raters/measurements, ICC3k - Two-way mixed effects, consistency, multiple raters/measurements. F – Fisher's F statistic, df – degrees of freedom.

	DunedinPACE						
	**ICC**	**F**	**df1**	**df2**	**P-value**	**Lower Bound**	**Upper Bound**
**ICC1**	-0.17	0.27	82	332	1.000	-0.19	-0.14
**ICC2**	0.03	2.28	82	328	<0.001	0.00	0.08
**ICC3**	0.20	2.28	82	328	<0.001	0.11	0.31
**ICC1k**	-2.74	0.27	82	332	1.000	-4.19	-1.61
**ICC2k**	0.13	2.28	82	328	<0.001	-0.01	0.29
**ICC3k**	0.56	2.28	82	328	<0.001	0.39	0.70

Table S6: Intraclass correlation coefficients across all tissues for the DunedinPACE estimates. ICC1 - One-way random effects, absolute agreement, single rater/measurement, ICC2 - Two-way random effects, absolute agreement, single rater/measurement, ICC3 - Two-way mixed effects, consistency, single rater/measurement, ICC1k - One-way random effects, absolute agreement, multiple raters/measurements, ICC2k - Two-way random effects, absolute agreement, multiple raters/measurements, ICC3k - Two-way mixed effects, consistency, multiple raters/measurements. F – Fisher's F statistic, df – degrees of freedom.

	Buccal vs Saliva		Buccal vs DBS		Buccal vs PBMC		Saliva vs DBS		Saliva vs PBMC		DBS vs PBMC	
	**Estimate**	**p-value**	**Estimate**	**p-value**	**Estimate**	**p-value**	**Estimate**	**p-value**	**Estimate**	**p-value**	**Estimate**	**p-value**
**Horvath**	-9.79	<0.001	-11.76	<0.001	-8.12	<0.001	-2.41	0.160	2.22	0.183	4.33	<0.001
**Hannum**	2.81	0.017	23.89	<0.001	28.26	<0.001	20.63	<0.001	24.16	<0.001	5.46	<0.001
**Skin and Blood**	-2.59	0.041	1.52	0.027	2.01	0.005	3.66	0.031	3.98	0.008	0.86	0.115
**PhenoAge**	-6.48	0.002	17.99	<0.001	27.10	<0.001	23.79	<0.001	32.75	<0.001	11.23	<0.001
**GrimAge2**	16.43	<0.001	35.89	<0.001	40.25	<0.001	17.45	<0.001	22.42	<0.001	5.16	<0.001
**DunedinPACE**	0.15	<0.001	0.66	<0.001	0.75	<0.001	0.47	<0.001	0.58	<0.001	0.1	<0.001

Table S7: Paired t-test results for within-person, between-tissue *adult* clock comparisons. This table mirrors **Table 2** from the manuscript. Positive values indicate a larger clock estimate for the tissue listed first in the tissue pair (i.e., a positive buccal vs. saliva value indicates buccal having a higher estimate than saliva).

	Buccal vs Saliva		Buccal vs DBS		Buccal vs Buffy Coat		Saliva vs DBS		Saliva vs Buffy Coat		DBS vs Buffy Coat	
	**Estimate**	**p-value**	**Estimate**	**p-value**	**Estimate**	**p-value**	**Estimate**	**p-value**	**Estimate**	**p-value**	**Estimate**	**p-value**
**Horvath**	-8.86	<0.001	-10.28	<0.001	-10.12	<0.001	-1.06	0.361	-1.69	0.196	-0.27	0.614
**Hannum**	-1.31	0.600	28.96	<0.001	29.29	<0.001	27.40	<0.001	29.28	<0.001	0.02	0.978
**Skin and Blood**	-0.76	0.255	1.10	<0.001	0.84	0.009	1.58	0.036	1.32	0.055	-0.29	0.123
**PhenoAge**	-3.35	0.160	24.56	<0.001	23.98	<0.001	25.71	<0.001	24.93	<0.001	-1.33	0.085
**GrimAge2**	8.39	<0.001	35.66	<0.001	34.82	<0.001	25.82	<0.001	25.70	<0.001	-1.11	0.181
**DunedinPACE**	0.01	0.905	0.63	<0.001	0.62	<0.001	0.62	<0.001	0.61	<0.001	-0.02	0.217

Table S8: Paired t-test results for within-person, between-tissue *children* clock comparisons. This table mirrors **Table 2** from the manuscript. Positive values indicate a larger clock estimate for the tissue listed first in the tissue pair (i.e., a positive buccal vs. saliva value indicates buccal having a higher estimate than saliva).

	Buccal vs Saliva		Buccal vs DBS		Buccal vs Buffy Coat		Buccal vs PBMC		Saliva vs DBS		Saliva vs Buffy Coat		Saliva vs PBMC		DBS vs Buffy Coat		DBS vs PBMC	
	**Est.**	**Pvalue**	**Est.**	**Pvalue**	**Est.**	**Pvalue**	**Est.**	**Pvalue**	**Est.**	**Pvalue**	**Est.**	**Pvalue**	**Est.**	**Pvalue**	**Est.**	**Pvalue**	**Est.**	**Pvalue**
**HorvathPC**	-11.41	<0.001	-18.90	<0.001	-13.56	<0.001	-18.21	<0.001	-8.12	<0.001	-8.54	<0.001	-2.38	0.110	-0.25	0.420	6.00	<0.001
**HannumPC**	7.54	<0.001	28.70	<0.001	32.55	<0.001	34.18	<0.001	19.65	<0.001	25.46	<0.001	24.73	<0.001	-0.11	0.800	9.54	<0.001
**Skin and Blood PC**	-6.60	<0.001	-10.34	<0.001	-10.56	<0.001	-1.51	0.190	-4.07	<0.001	-5.27	<0.001	5.76	<0.001	-0.49	0.290	9.33	<0.001
**PhenoAgePC**	14.31	<0.001	59.60	<0.001	64.13	<0.001	68.60	<0.001	42.51	<0.001	50.03	<0.001	50.68	<0.001	-0.64	0.360	14.41	<0.001
**GrimAgePC**	7.75	<0.001	20.01	<0.001	20.58	<0.001	25.24	<0.001	11.42	<0.001	13.53	<0.001	16.22	<0.001	-0.61	0.038	6.67	<0.001

Table S9: Paired t-test results for within-person, between-tissue PC clock comparisons. This table mirrors **Table 2** from the manuscript. Positive values indicate a larger clock estimate for the tissue listed first in the tissue pair (i.e., a positive buccal vs. saliva value indicates buccal having a higher estimate than saliva).

	Buccal		Saliva		DBS		Buffy Coat		PBMC	
	**Estimate**	**p-value**	**Estimate**	**p-value**	**Estimate**	**p-value**	**Estimate**	**p-value**	**Estimate**	**p-value**
**HorvathPC**	13.11	<0.001	11.63	<0.001	4.70	<0.001	2.59	<0.001	8.04	<0.001
**HannumPC**	-27.14	<0.001	-20.94	<0.001	-24.54	<0.001	-24.57	<0.001	-20.55	<0.001
**Skin and Blood PC**	-3.45	<0.001	-8.34	<0.001	-15.37	<0.001	-17.01	<0.001	-5.25	<0.001
**PhenoAgePC**	-40.11	<0.001	-19.91	<0.001	-1.74	0.005	-0.94	0.160	1.71	0.042
**GrimAgePC**	7.39	<0.001	1.52	0.084	-8.00	<0.001	-6.97	<0.001	-6.88	<0.001

Table S10: Paired t-test results comparing the standard and PC clock estimates within-person and within-tissue. Positive values indicate a larger standard clock estimate and negative values indicate a larger PC clock estimate.

S2.2. Estimation of Tissue Cellular Composition

Figure S1 shows the results from using the Houseman-ReferenceFree method on both buccal and saliva tissue to estimate the "best-fitting" number of cell subtypes. 1000 bootstrapped samples composed of around 36.8% of the original sample were drawn randomly from the tissue of interest (with replacement) and deviance statistics were calculated for each sample using an assumed 1-15 cell subtypes. Code for the calculation of deviance statistics is located at https://github.com/abnerapsley1/CrossTissueEpiClock.

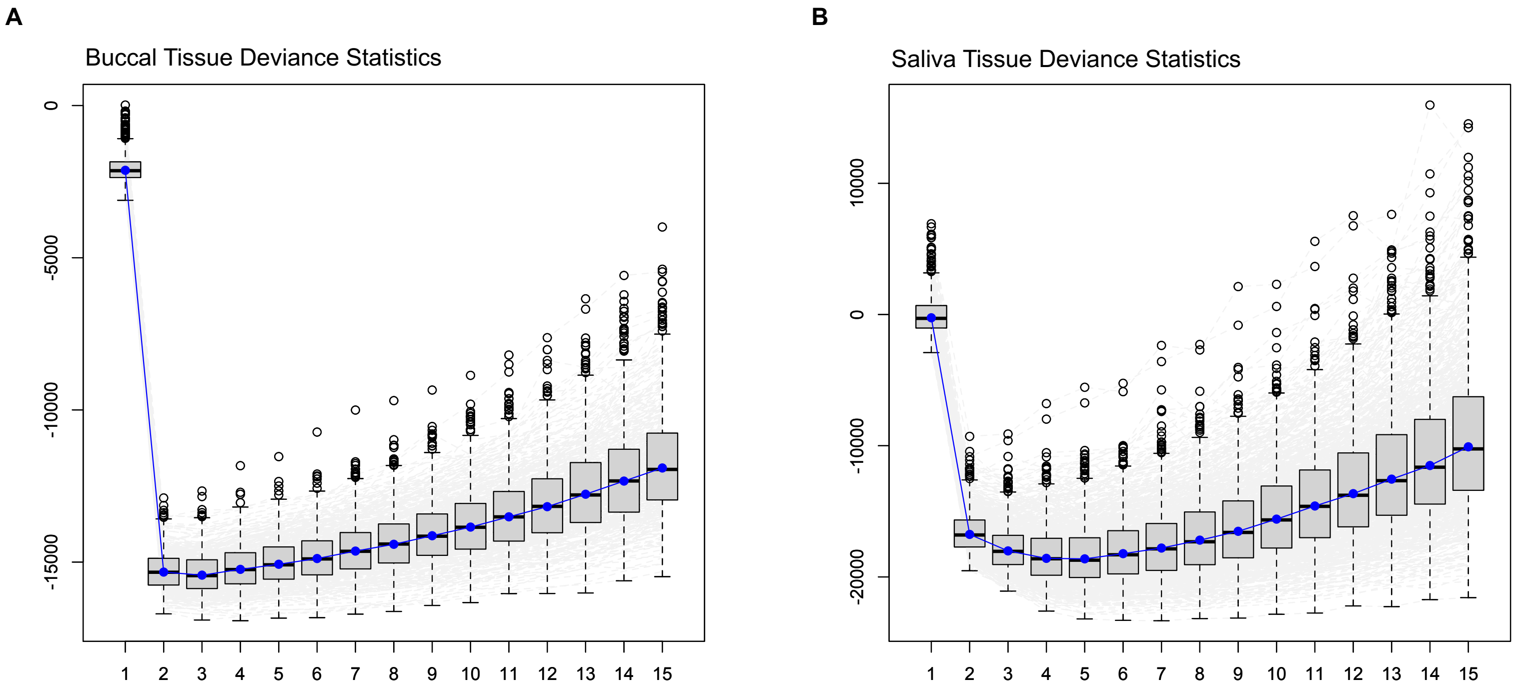

Figure S1 – Houseman-ReferenceFree Method Buccal (A) and Saliva (B) Deviance Statistics. X-axes show the number of estimated cell types and y-axes show the deviance statistic. Blue dots indicate the median deviance statistic, grey boxes indicate upper and lower quartiles and whiskers indicate 95th percentiles. An estimation of three cell types best fit the buccal tissue (A) and five cell types best fit the saliva tissue (B).

S2.3. Comparability of First-Generation Epigenetic Clocks Across Tissues – Stratified by Age

Figure S2 parallels **Figure 1** from the main text, however only adults (18 years or older) are included in the sample. Similarly, **Figure S3** parallels **Figure 1** from the main text but only includes children (younger than 18 years old).

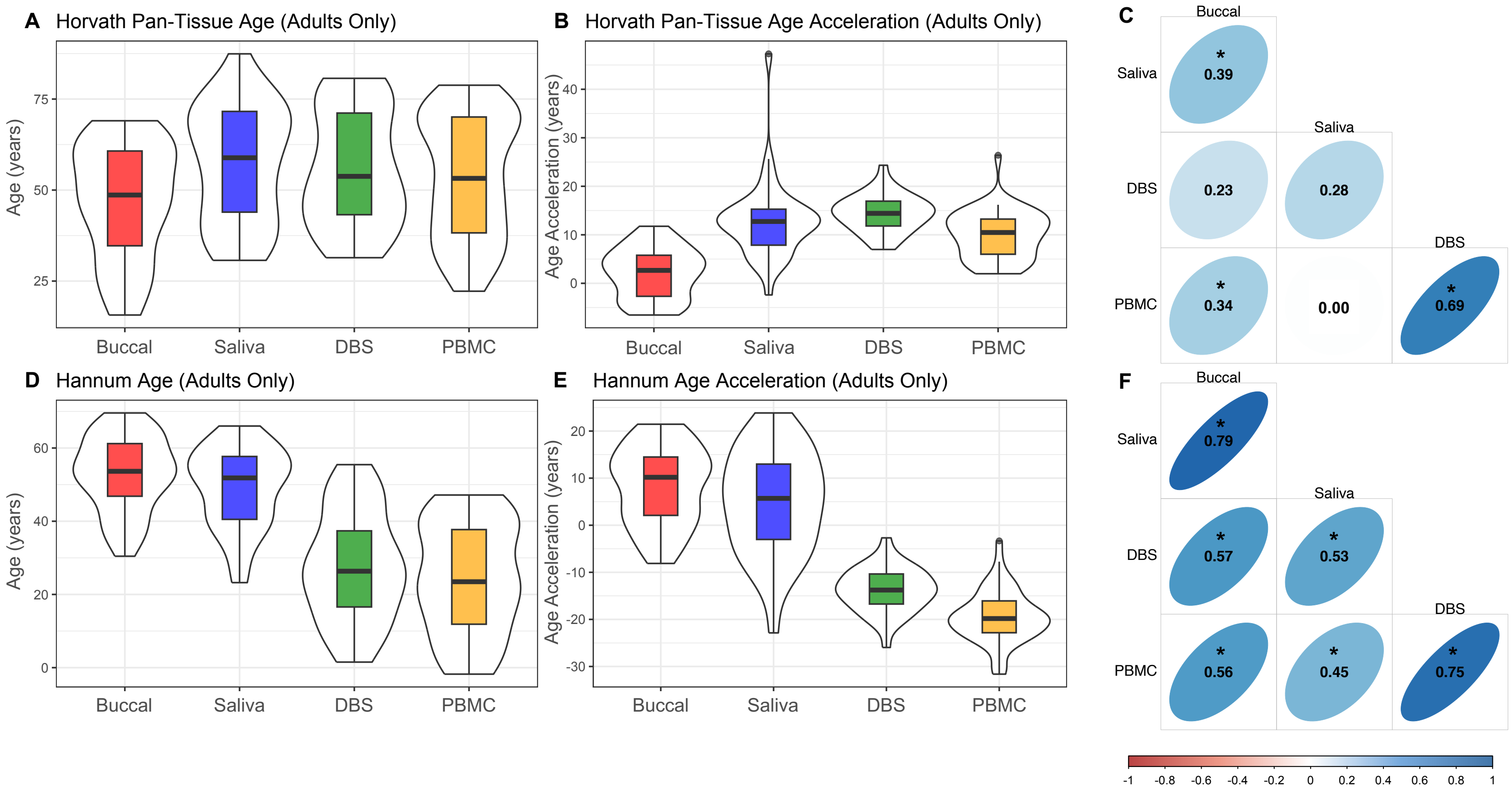

Figure S2 – First Generation Epigenetic Clock Distributions and Correlations (Adults Only).
(A) Horvath pan-tissue epigenetic age estimates for buccal (46.69 ± 15.72), saliva (56.48 ± 16.39), DBS (55.75 ± 15.84), and PBMCs (53.74 ± 16.87), (B) Horvath pan-tissue epigenetic age acceleration estimates for buccal (2.07 ± 5.23), saliva (12.23 ± 8.46), DBS (14.50 ± 3.87), and PBMCs (9.99 ± 4.95), (C) Within-person Horvath pan-tissue epigenetic age acceleration correlations across

tissues, (D) Hannum epigenetic age estimates for buccal (52.77 ± 10.18), saliva (48.94 ± 10.97), DBS (27.49 ± 14.83), and PBMCs (24.27 ± 15.12), (E) Hannum epigenetic age acceleration estimates for buccal (8.15 ± 8.22), saliva (4.68 ± 11.02), DBS (-13.76 ± 4.68), and PBMCs (-19.47 ± 5.57) and (F) Within-person Hannum epigenetic age acceleration correlations across tissues. Thick black horizontal bars on violin plots indicate the tissue-stratified median clock value and colored boxes indicate interquartile ranges. * indicate correlations with $p<0.05$.

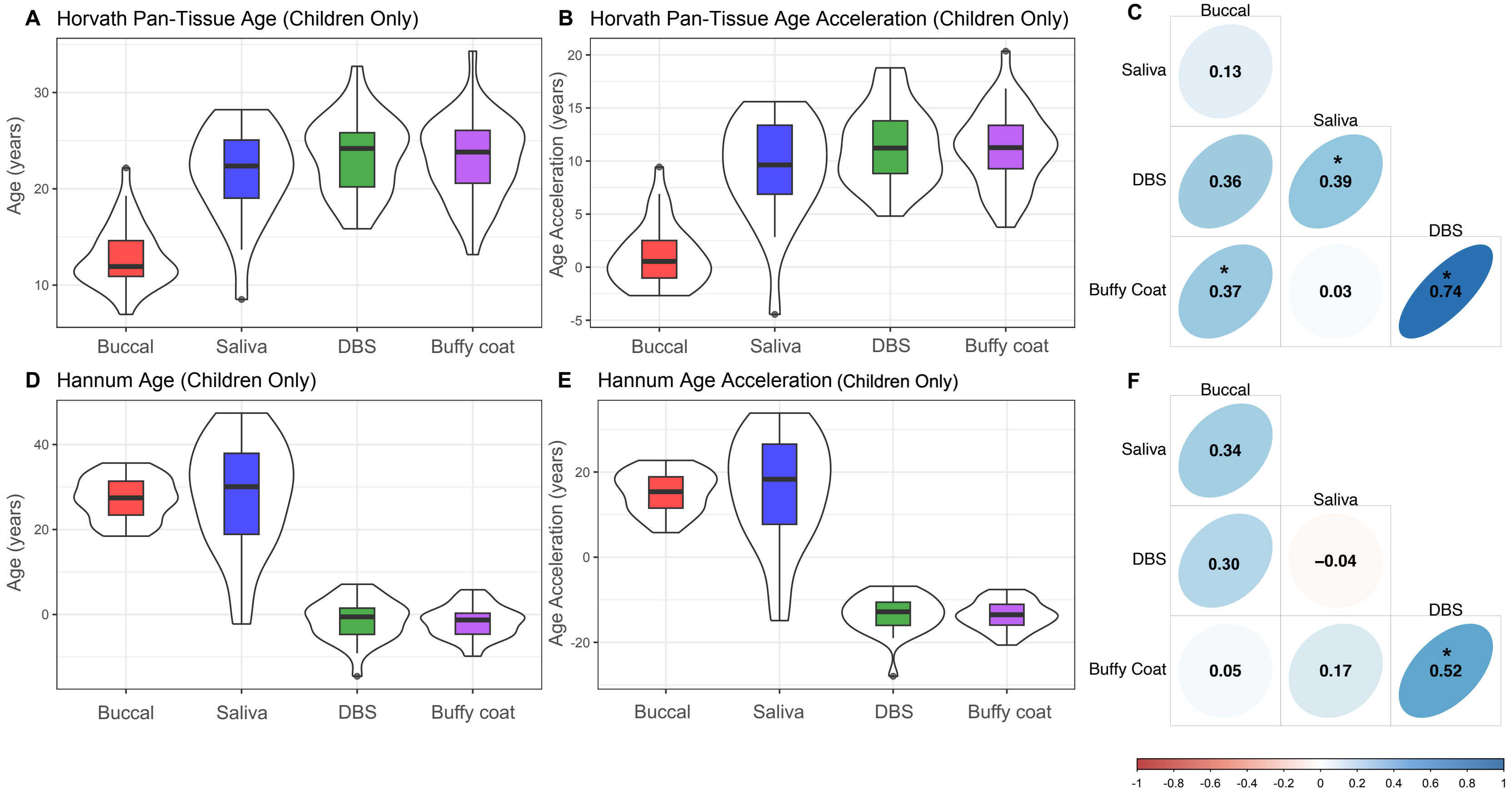

Figure S3 – First Generation Epigenetic Clock Distributions and Correlations (Children Only).
(A) Horvath pan-tissue epigenetic age estimates for buccal (12.89 ± 3.24), saliva (21.50 ± 4.81), DBS (23.48 ± 4.04), and buffy coat (23.12 ± 4.43), (B) Horvath pan-tissue epigenetic age acceleration estimates for buccal (0.97 ± 2.9), saliva (9.33 ± 4.71), DBS (11.44 ± 3.70), and buffy coat (11.21 ± 3.81), (C) Within-person Horvath pan-tissue epigenetic age acceleration correlations across tissues, (D) Hannum epigenetic age estimates for buccal (27.29 ± 5.02), saliva (28.09 ± 12.99), DBS (-1.27 ± 4.76), and buffy coat (-1.72 ± 3.83), (E) Hannum epigenetic age acceleration estimates for buccal (15.37 ± 4.72), saliva (15.92 ± 12.92), DBS (-13.32 ± 4.40), and buffy coat (-13.64 ± 3.39) and (F) Within-person Hannum epigenetic age acceleration correlations across tissues. Thick black

horizontal bars on violin plots indicate the tissue-stratified median clock value and colored boxes indicate interquartile ranges. * indicate correlations with $p<0.05$.

S2.4. Comparability of Skin/Blood and PedBE Epigenetic Clocks Across Tissues – Stratified by Age

Figure S4 parallels **Figure 2** from the main text, however only adults (18 years or older) are included in the sample and the PedBE clock is not included. Similarly, **Figure S5** parallels **Figure 2** from the main text but only includes children (younger than 18 years old) and does not include the PedBE clock.

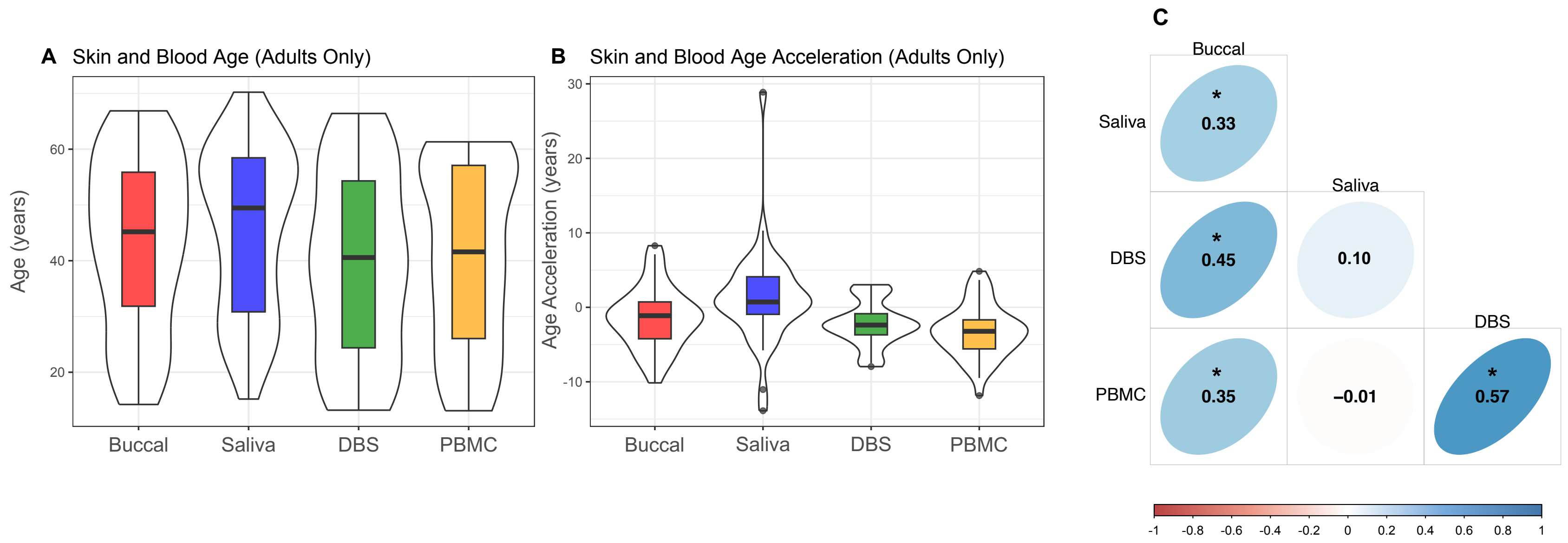

Figure S4 – Skin/Blood Clock Distributions and Correlations (Adults Only).
(A) Skin/Blood epigenetic age estimates for buccal (43.07 ± 15.53), saliva (44.95 ± 15.37), DBS (39.04 ± 16.65), and PBMCs (40.48 ± 16.25), (B) Skin/Blood epigenetic age acceleration estimates for buccal (-1.55 ± 4.34), saliva (0.69 ± 7.46), DBS (-2.21 ± 3.01), and PBMCs (-3.26 ± 3.53), (C) Within-person Skin/Blood epigenetic age acceleration correlations across tissues. Thick black horizontal bars on violin plots indicate the tissue-stratified median clock value and colored boxes indicate interquartile ranges. * indicate correlations with $p<0.05$.

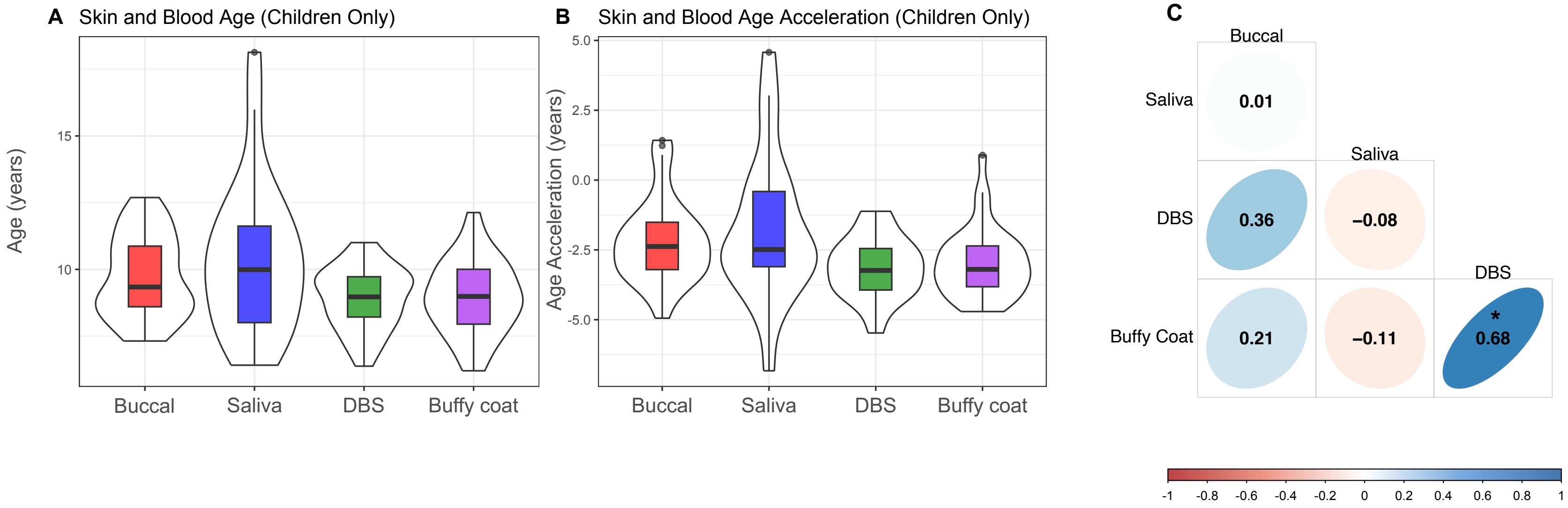

Figure S5 – Skin/Blood Clock Distributions and Correlations (Children Only).
(A) Skin/Blood epigenetic age estimates for buccal (9.71 ± 1.50), saliva (10.46 ± 2.88), DBS (8.92 ± 1.12), and buffy coat (8.96 ± 1.42), (B) Skin/Blood epigenetic age acceleration estimates for buccal (-2.21 ± 1.45), saliva (-1.71 ± 2.64), DBS (-3.12 ± 1.05), and buffy coat (-2.96 ± 1.25), (C) Within-person Skin/Blood epigenetic age acceleration correlations across tissues. Thick black horizontal bars on violin plots indicate the tissue-stratified median clock value and colored boxes indicate interquartile ranges. * indicate correlations with $p<0.05$.

S2.5. Comparability of Second-Generation Epigenetic Clocks Across Tissues – Stratified by Age

Figure S6 parallels **Figure 3** from the main text, however only adults (18 years or older) are included in the sample. Similarly, **Figure S7** parallels **Figure 3** from the main text but only includes children (younger than 18 years old).

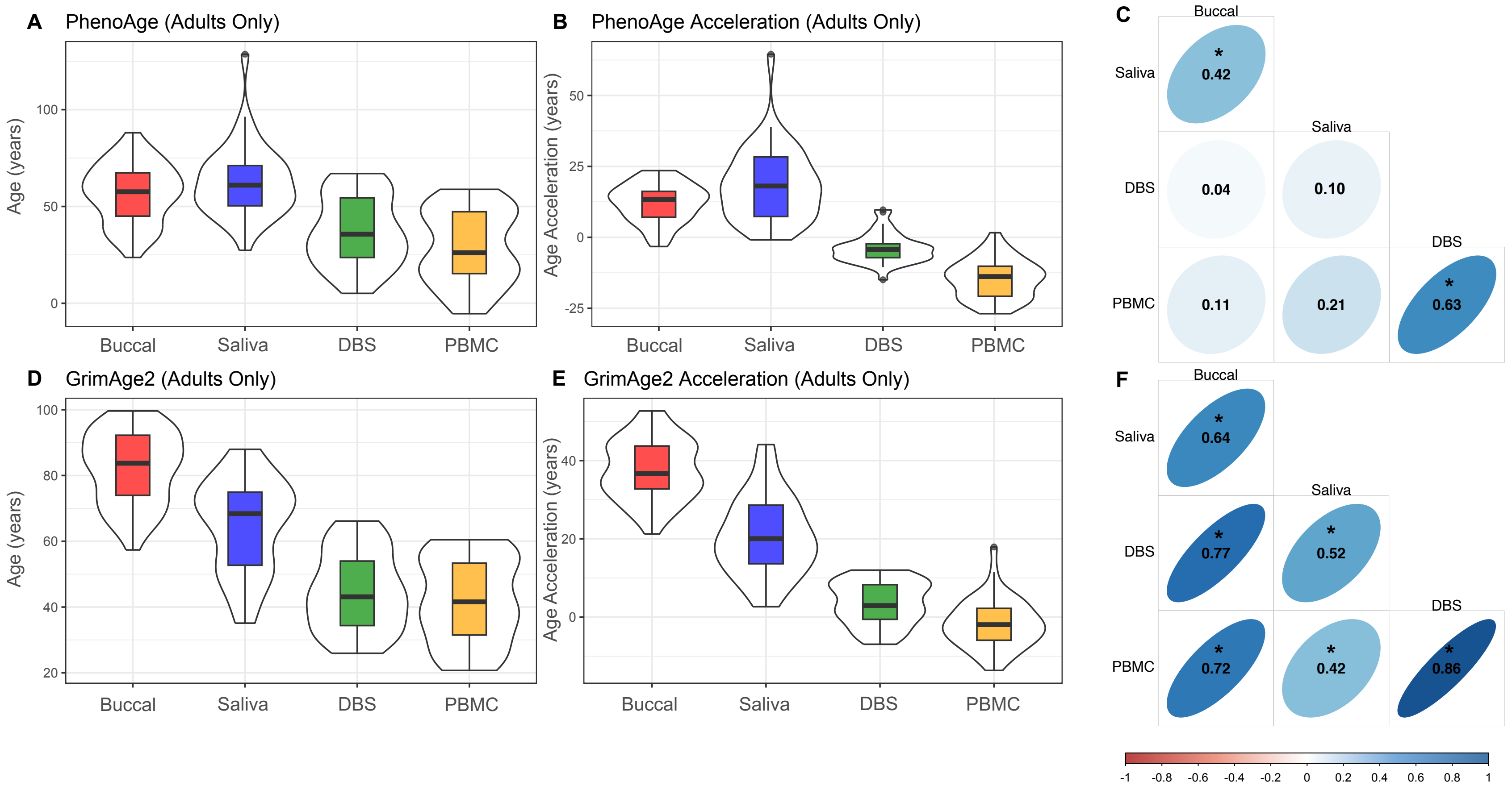

Figure S6 – Second Generation Epigenetic Clock Distributions and Correlations (Adults Only).
(A) PhenoAge epigenetic age estimates for buccal (56.77 ± 15.12), saliva (62.71 ± 18.67), DBS (37.45 ± 17.91), and PBMCs (29.03 ± 18.91), (B) PhenoAge epigenetic age acceleration estimates for buccal (12.15 ± 7.04), saliva (18.46 ± 12.77), DBS (-3.81 ± 5.21), and

PBMCs (-14.71 ± 6.68), (C) Within-person PhenoAge epigenetic age acceleration correlations across tissues, (D) GrimAge2 epigenetic age estimates for buccal (82.27 ± 11.15), saliva (65.00 ± 14.12), DBS (44.90 ± 11.88), and PBMCs (42.01 ± 12.73), (E) GrimAge2 epigenetic age acceleration estimates for buccal (37.65 ± 7.63), saliva (20.74 ± 10.12), DBS (3.65 ± 5.49), and PBMCs (-1.74 ± 6.15) and (F) Within-person GrimAge2 epigenetic age acceleration correlations across tissues. Thick black horizontal bars on violin plots indicate the tissue-stratified median clock value and colored boxes indicate interquartile ranges. * indicate correlations with $p<0.05$.

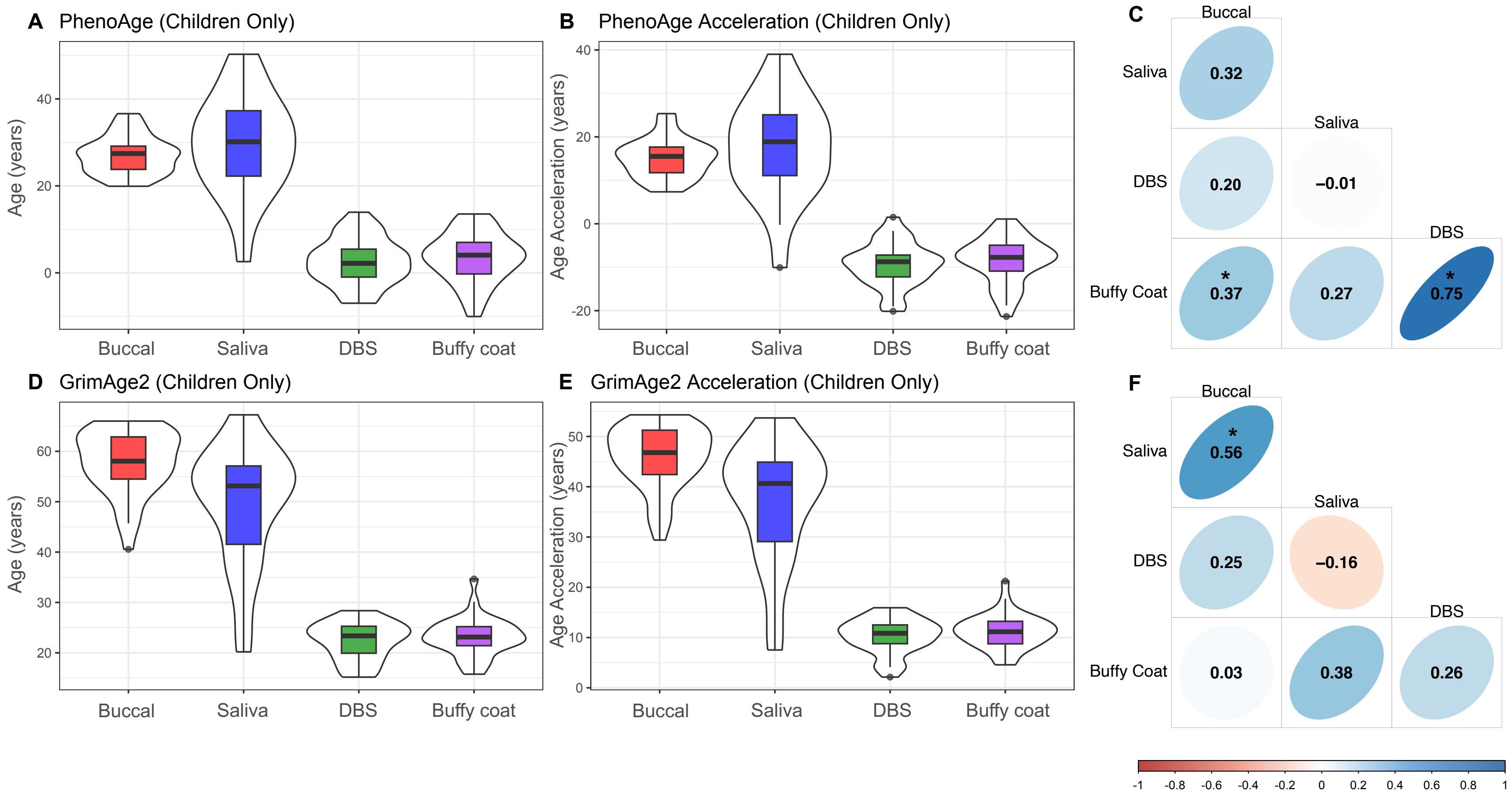

Figure S7 - Second Generation Epigenetic Clock Distributions and Correlations (Children Only).
(A) PhenoAge epigenetic age estimates for buccal (27.08 ± 4.30), saliva (29.43 ± 11.53), DBS (2.71 ± 5.73), and buffy coat (3.43 ± 5.74), (B) PhenoAge epigenetic age acceleration estimates for buccal (15.16 ± 4.39), saliva (17.25 ± 11.63), DBS (-9.34 ± 5.49), and buffy coat (-8.48 ± 5.30), (C) Within-person PhenoAge epigenetic age acceleration correlations across tissues, (D) GrimAge2 epigenetic age estimates for buccal (57.77 ± 6.28), saliva (49.37 ± 11.89), DBS (22.35 ± 3.73), and buffy coat (23.10 ± 3.84), (E) GrimAge2 epigenetic age acceleration estimates for buccal (45.85 ± 6.17), saliva (37.20 ± 11.83), DBS (10.31 ± 3.49), and buffy coat

(11.19 ± 3.41) and (F) Within-person GrimAge2 epigenetic age acceleration correlations across tissues. Thick black horizontal bars on violin plots indicate the tissue-stratified median clock value and colored boxes indicate interquartile ranges. * indicate correlations with $p<0.05$.

S2.6. Comparability of DunedinPACE Epigenetic Clock Across Tissues – Stratified by Age

Figure S8 parallels **Figure 4** from the main text, however only adults (18 years or older) are included in the sample. Similarly, **Figure S9** parallels **Figure 4** from the main text but only includes children (younger than 18 years old).

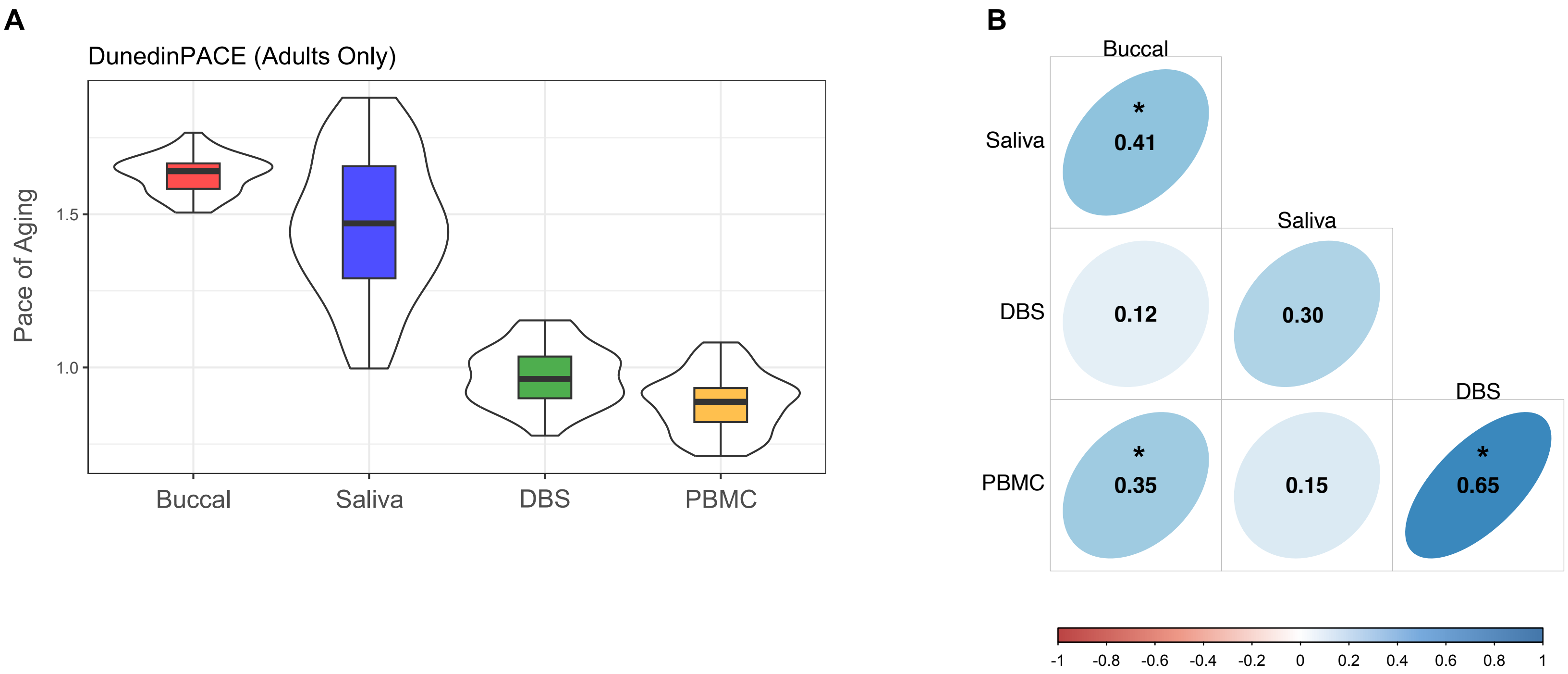

Figure S8 - DunedinPace Epigenetic Clock Distributions and Correlations (Adults Only).
(A) DunedinPACE estimates for buccal (1.63 ± 0.06), saliva (1.46 ± 0.24), DBS (0.97 ± 0.10), and PBMCs (0.89 ± 0.10), (B) Within-person DunedinPACE estimate correlations across tissues. Thick black horizontal bars on violin plots indicate the tissue-stratified median clock value and colored boxes indicate interquartile ranges. * indicate correlations with $p<0.05$.

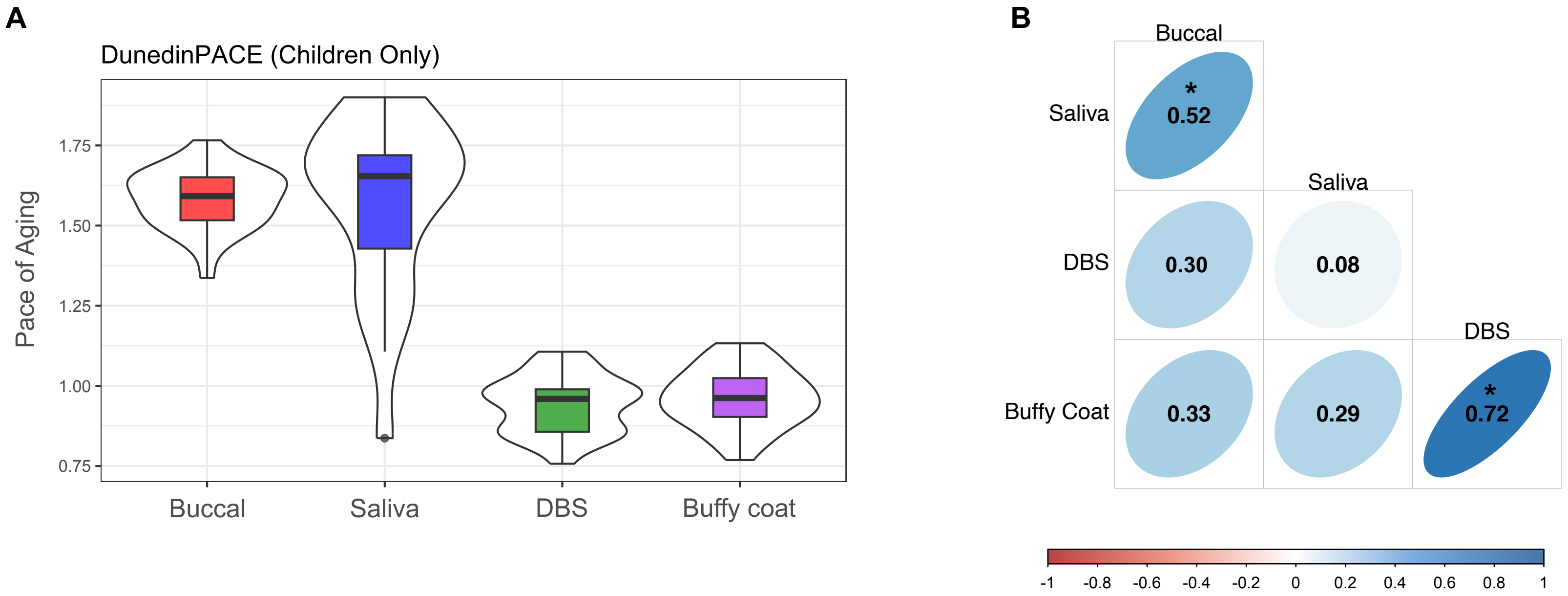

Figure S9 - DunedinPace Epigenetic Clock Distributions and Correlations (Children Only).
(A) DunedinPACE estimates for buccal (1.58 ± 0.09), saliva (1.57 ± 0.27), DBS (0.94 ± 0.09), and buffy coat (0.96 ± 0.09), (B) Within-person DunedinPACE estimate correlations across tissues. Thick black horizontal bars on violin plots indicate the tissue-stratified median clock value and colored boxes indicate interquartile ranges. * indicate correlations with $p<0.05$.

S2.7. Sensitivity Analyses – PC Clocks (Full Sample and Stratified by Age)

The following figures are full sample (**Figures S10-S12**) and age-stratified results (**Figures S13-S18**) of principal component clocks.

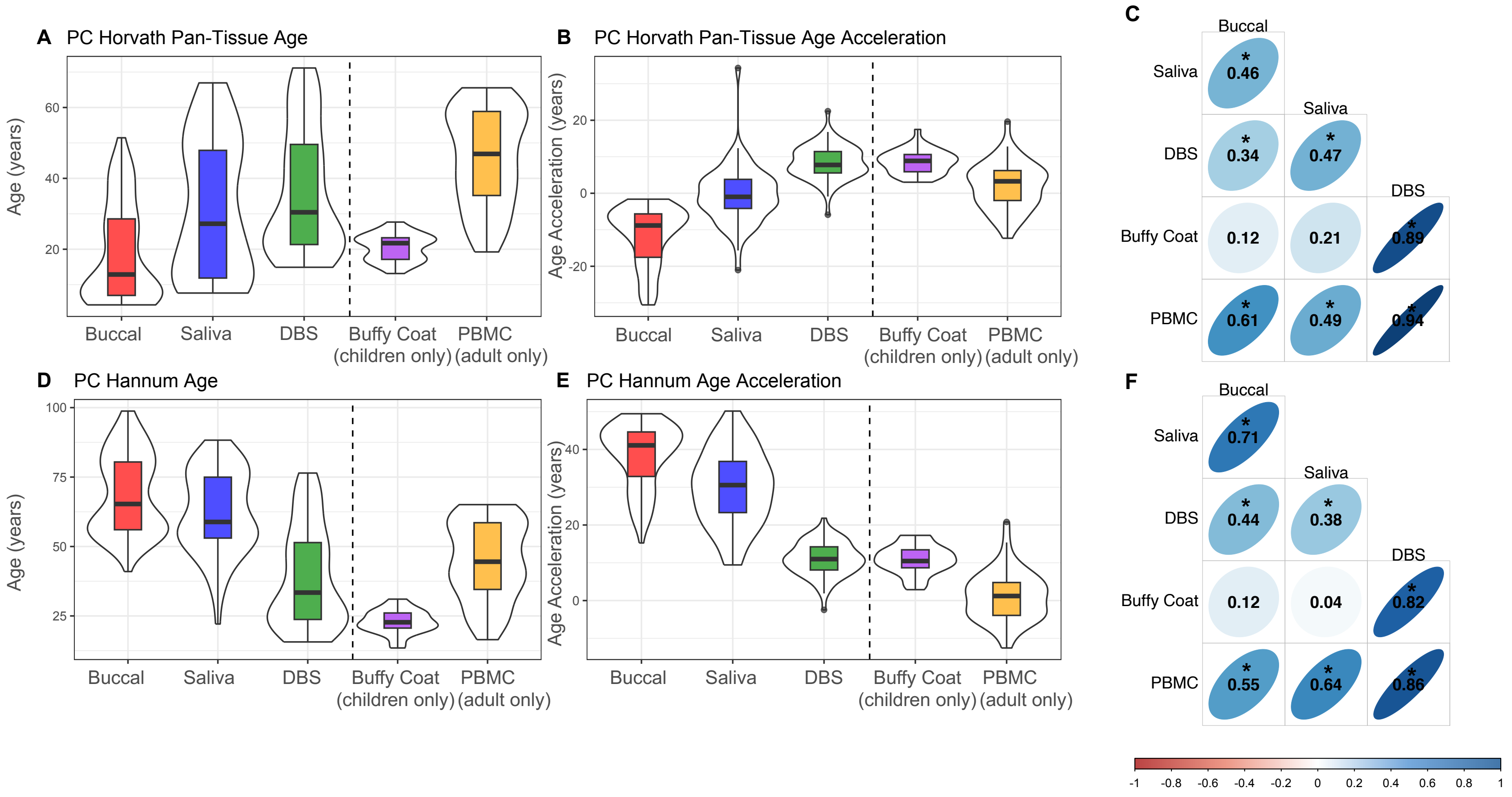

Figure S10 – First Generation Principal Component Epigenetic Clock Distributions and Correlations.
(A) PC-Horvath pan-tissue epigenetic age estimates for buccal (18.97 ± 13.74), saliva (31.81 ± 19.34), DBS (36.47 ± 17.77), buffy coat (20.54 ± 3.74), and PBMC (45.69 ± 13.59), (B) PC-Horvath pan-tissue epigenetic age acceleration estimates for buccal (-11.52 ± 8.03), saliva (-0.48 ± 8.04), DBS (8.46 ± 4.64), buffy coat (8.62 ± 3.24), and PBMC (1.95 ± 6.49), (C) Within-person PC-Horvath pan-tissue epigenetic age acceleration correlations across tissues, (D) PC-Hannum epigenetic age estimates for buccal (68.90 ± 14.39),

saliva (62.10 ± 14.85), DBS (39.00 ± 17.88), buffy coat (22.85 ± 4.27), and PBMC (44.83 ± 14.00), (E) PC-Hannum epigenetic age acceleration estimates for buccal (38.41 ± 8.48), saliva (29.81 ± 9.90), DBS (10.98 ± 4.46), buffy coat (10.94 ± 3.79), and PBMC (1.08 ± 6.53) and (F) Within-person PC-Hannum epigenetic age acceleration correlations across tissues. Thick black horizontal bars on violin plots indicate the tissue-stratified median clock value and colored boxes indicate interquartile ranges. * indicate correlations with $p<0.05$.

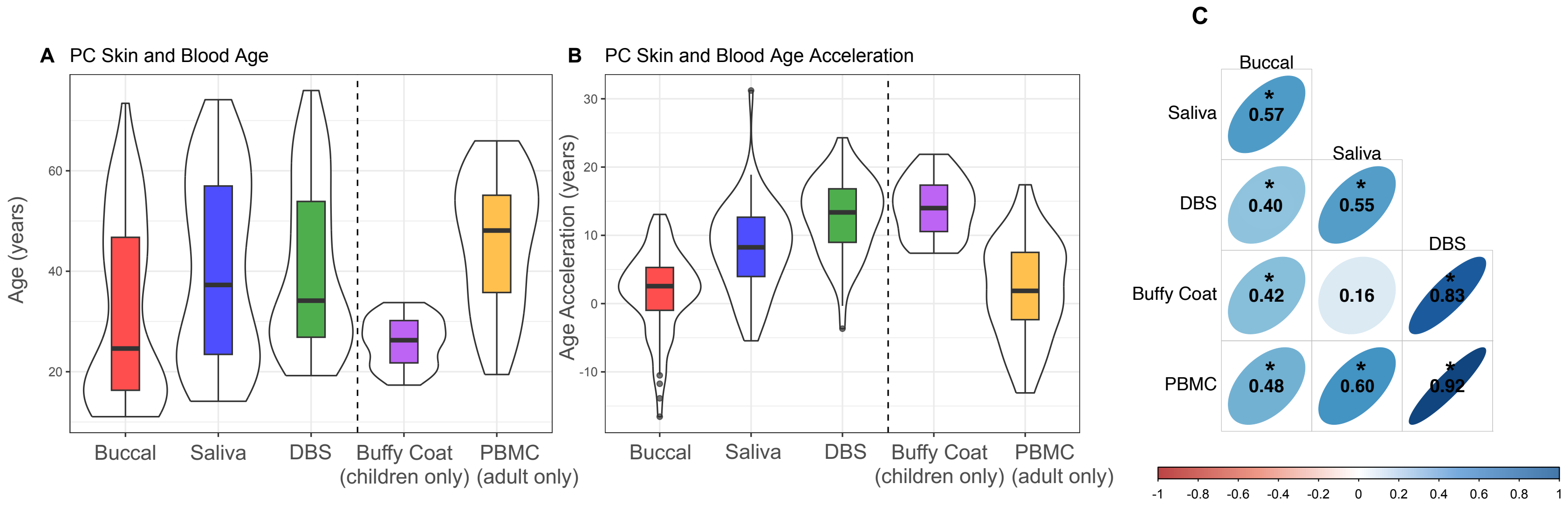

Figure S11 – Skin and Blood Principal Component Epigenetic Clock Distributions and Correlations.
(A) PC-SkinBlood epigenetic age estimates for buccal (32.11 ± 18.04), saliva (40.43 ± 18.30), DBS (40.76 ± 16.61), buffy coat (25.97 ± 4.76), and PBMC (45.73 ± 13.30), (B) PC-SkinBlood epigenetic age acceleration estimates for buccal (1.62 ± 6.05), saliva (8.13 ± 6.55), DBS (12.74 ± 5.65), buffy coat (14.06 ± 4.24), and PBMC (1.99 ± 7.30), (C) Within-person PC-SkinBlood epigenetic age acceleration correlations across tissues. Thick black horizontal bars on violin plots indicate the tissue-stratified median clock value and colored boxes indicate interquartile ranges. * indicate correlations with $p<0.05$.

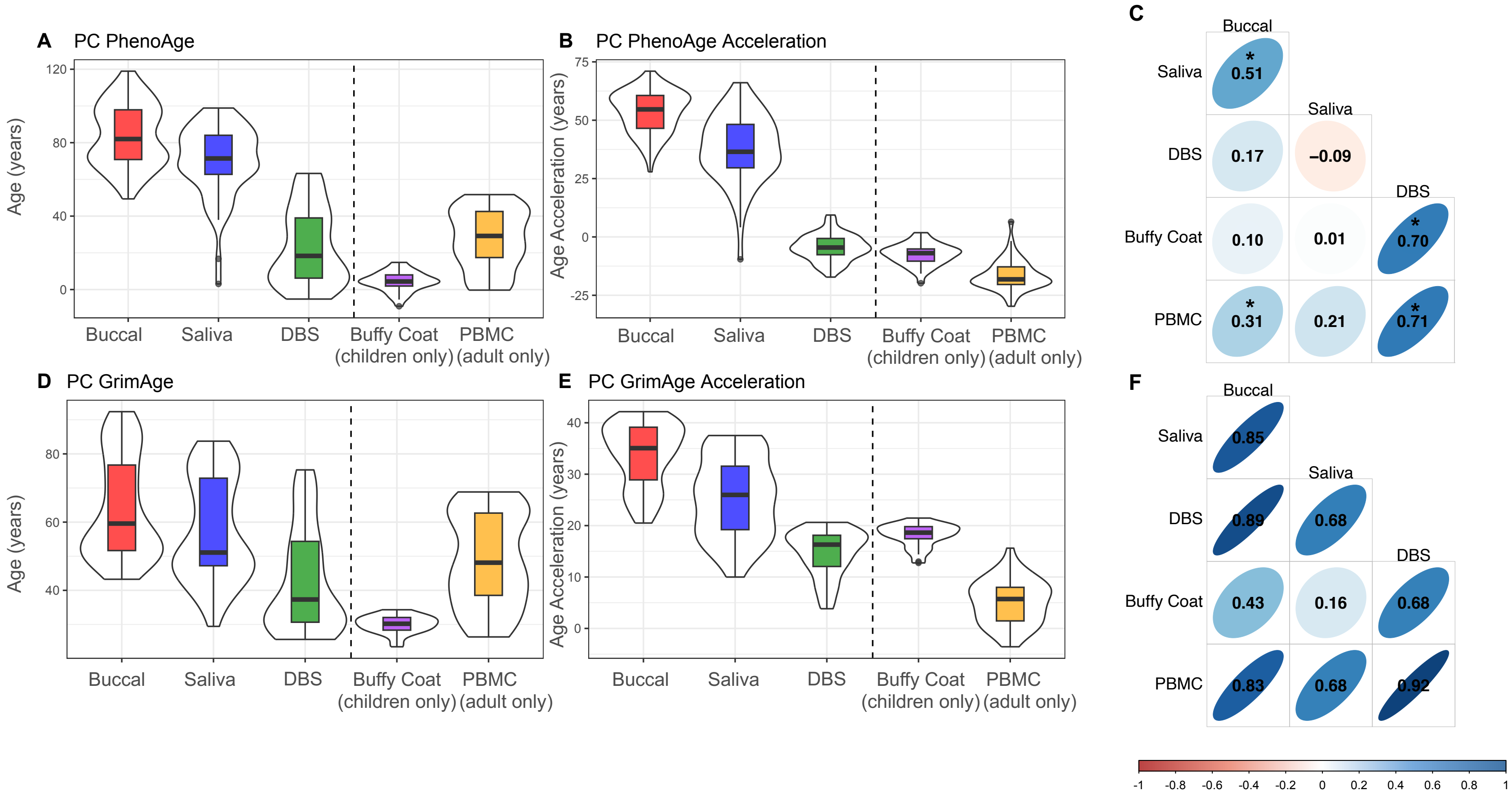

Figure S12 – Second Generation Principal Component Epigenetic Clock Distributions and Correlations. (A) PC-PhenoAge epigenetic age estimates for buccal (84.05 ± 16.97), saliva (70.21 ± 19.24), DBS (23.44 ± 20.94), buffy coat (4.38 ± 5.41), and PBMC (27.33 ± 16.23), (B) PC-PhenoAge epigenetic age acceleration estimates for buccal (53.56 ± 9.04), saliva (37.92 ± 14.53), DBS (-4.57 ± 5.67), buffy coat (-7.54 ± 4.68), and PBMC (-16.42 ± 6.55), (C) Within-person PC-PhenoAge epigenetic age acceleration correlations across tissues, (D) PC-GrimAge epigenetic age estimates for buccal (64.29 ± 14.93), saliva (57.66 ± 14.94), DBS (42.68 ± 15.19), buffy coat (30.07 ± 2.62), and PBMC (48.89 ± 13.55), (E) PC-GrimAge epigenetic age acceleration estimates

for buccal (33.80 ± 6.18), saliva (25.36 ± 7.71), DBS (14.67 ± 4.49), buffy coat (18.16 ± 2.20), and PBMC (5.15 ± 4.36) and (F) Within-person PC-GrimAge epigenetic age acceleration correlations across tissues. Thick black horizontal bars on violin plots indicate the tissue-stratified median clock value and colored boxes indicate interquartile ranges. * indicate correlations with $p<0.05$.

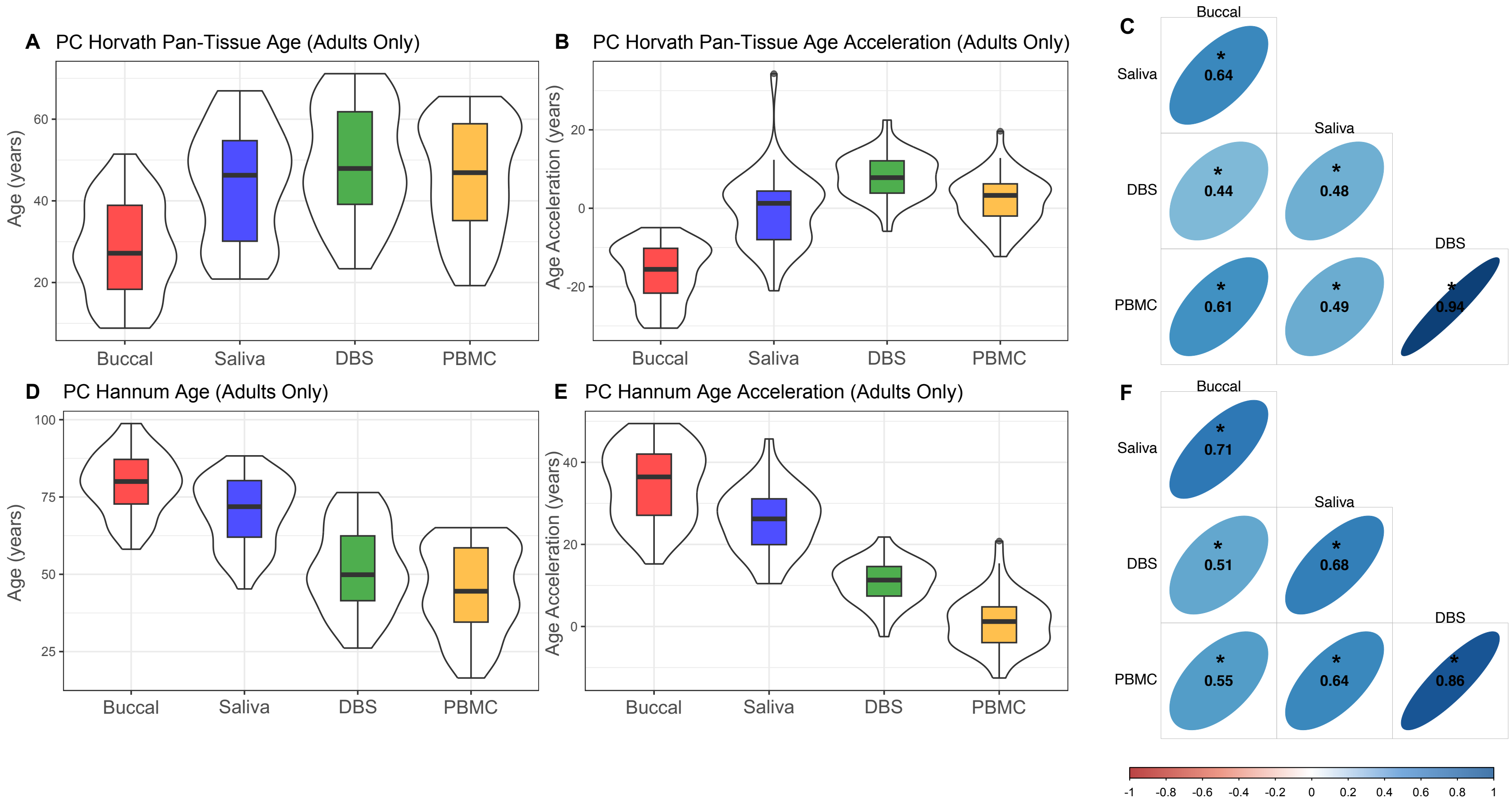

Figure S13 – First Generation Principal Component Epigenetic Clock Distributions and Correlations (Adults Only). (A) PC-Horvath pan-tissue epigenetic age estimates for buccal (28.15 ± 11.61), saliva (43.80 ± 14.05), DBS (49.42 ± 13.93), and PBMCs (45.69 ± 13.59), (B) PC-Horvath pan-tissue epigenetic age acceleration estimates for buccal (-16.47 ± 7.36), saliva (-0.46 ± 9.75), DBS (8.17 ± 5.60), and PBMCs (1.95 ± 6.49), (C) Within-person PC-Horvath pan-tissue epigenetic age acceleration correlations across tissues, (D) PC-Hannum epigenetic age estimates for buccal (79.35 ± 9.86), saliva (70.19 ± 11.04), DBS (52.03 ± 13.90), and PBMCs (44.83 ± 14.00), (E) PC-Hannum epigenetic age acceleration estimates for buccal (34.73 ± 9.18), saliva (25.93 ±

7.90), DBS (10.78 ± 5.16), and PBMCs (1.08 ± 6.53) and (F) Within-person PC-Hannum epigenetic age acceleration correlations across tissues. Thick black horizontal bars on violin plots indicate the tissue-stratified median clock value and colored boxes indicate interquartile ranges. * indicate correlations with $p<0.05$.

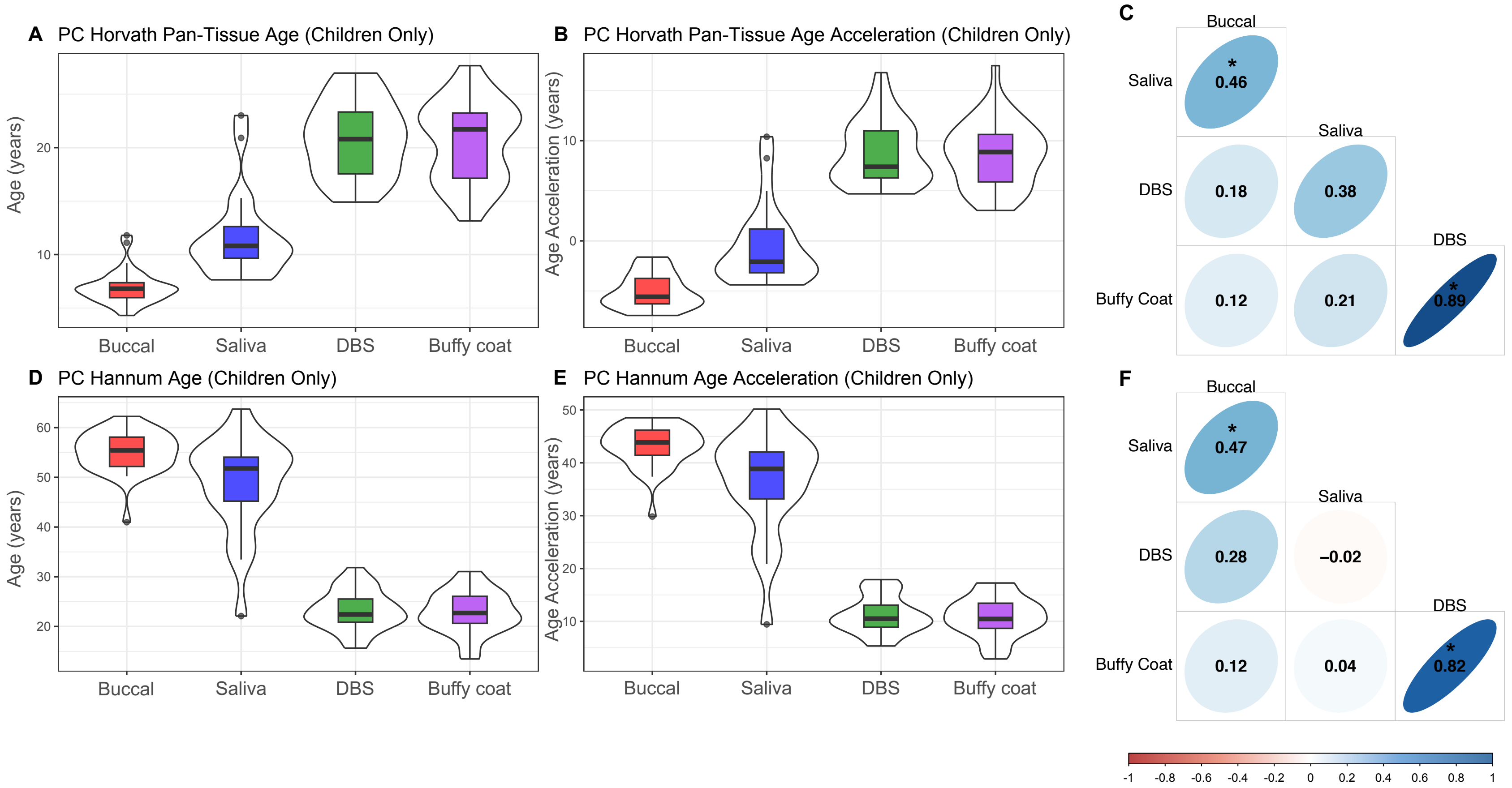

Figure S14 – First Generation Principal Component Epigenetic Clock Distributions and Correlations (Children Only). (A) PC-Horvath pan-tissue epigenetic age estimates for buccal (6.91 ± 1.55), saliva (11.65 ± 3.97), DBS (20.85 ± 3.50), and buffy coat (20.54 ± 3.74), (B) PC-Horvath pan-tissue epigenetic age acceleration estimates for buccal (-5.01 ± 1.74), saliva (-0.53 ± 3.96), DBS (8.80 ± 3.19), and buffy coat (8.62 ± 3.24), (C) Within-person PC-Horvath pan-tissue epigenetic age acceleration correlations across tissues, (D) PC-Hannum epigenetic age estimates for buccal (55.16 ± 4.06), saliva (48.51 ± 9.59), DBS (23.27 ± 3.99), and buffy coat (22.85 ± 4.27), (E) PC-Hannum epigenetic age acceleration estimates for buccal (43.25 ± 3.91), saliva (36.34 ± 9.61), DBS (11.23 ± 3.52), and buffy coat (10.94 ± 3.79) and (F) Within-person PC-Hannum epigenetic age acceleration correlations across tissues. Thick

black horizontal bars on violin plots indicate the tissue-stratified median clock value and colored boxes indicate interquartile ranges. * indicate correlations with $p<0.05$.

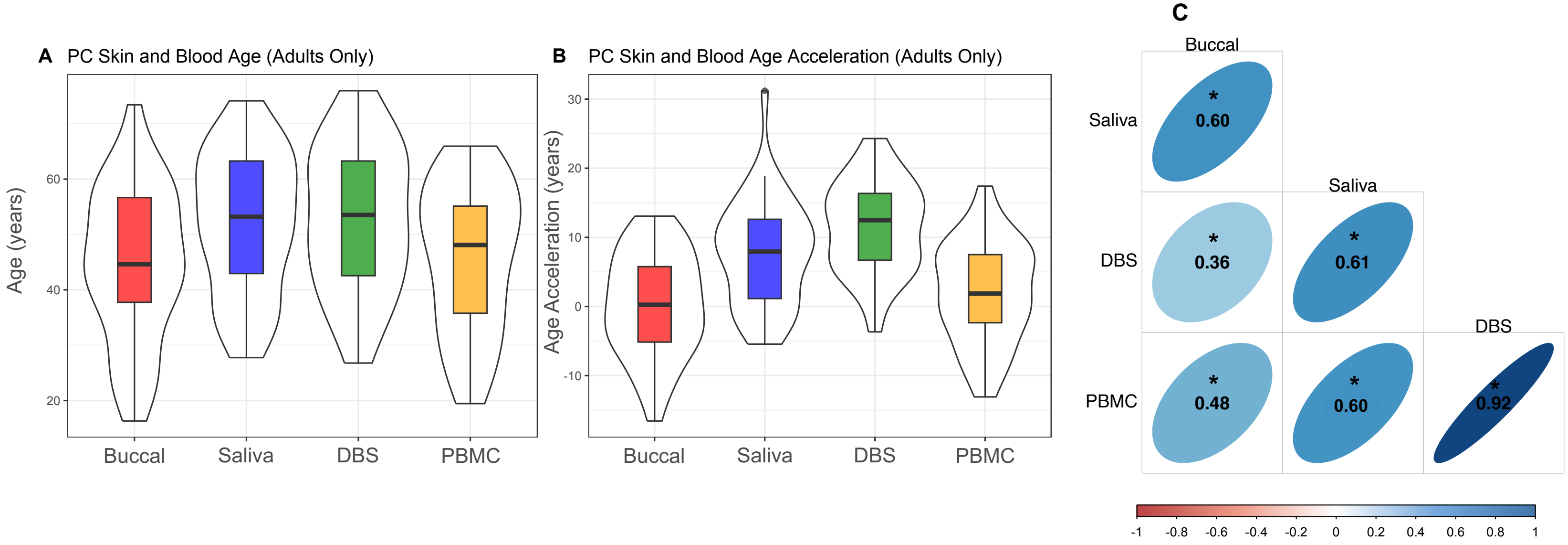

Figure S15 – Skin and Blood Principal Component Epigenetic Clock Distributions and Correlations (Adults Only). (A) PC-SkinBlood epigenetic age estimates for buccal (44.80 ± 13.79), saliva (52.00 ± 12.65), DBS (52.70 ± 13.12), and PBMCs (45.73 ± 13.30), (B) PC-SkinBlood epigenetic age acceleration estimates for buccal (0.18 ± 7.42), saliva (7.74 ± 7.74), DBS (11.45 ± 6.62), and PBMCs (1.99 ± 7.30), (C) Within-person PC-SkinBlood epigenetic age acceleration correlations across tissues. Thick black horizontal bars on violin plots indicate the tissue-stratified median clock value and colored boxes indicate interquartile ranges. * indicate correlations with $p<0.05$.

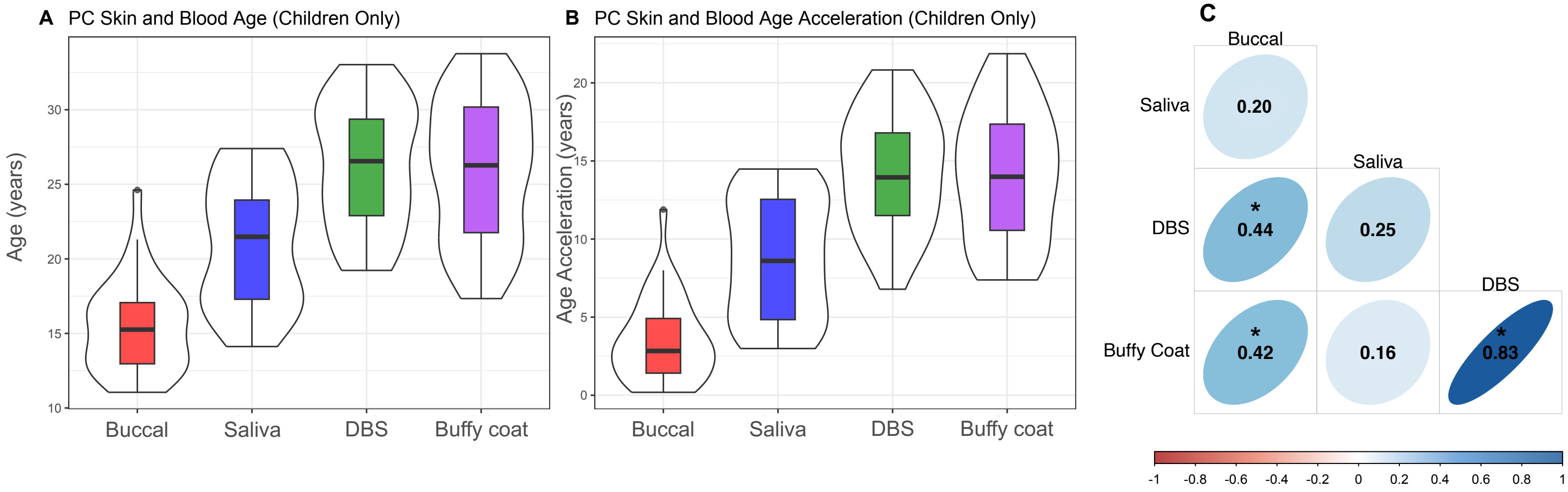

Figure S16 – Skin and Blood Principal Component Epigenetic Clock Distributions and Correlations (Children Only). (A) PC-SkinBlood epigenetic age estimates for buccal (15.42 ± 3.07), saliva (20.97 ± 4.24), DBS (26.34 ± 4.23), and buffy coat (25.97 ± 4.76), (B) PC-SkinBlood epigenetic age acceleration estimates for buccal (3.50 ± 2.63), saliva (8.79 ± 3.91), DBS (14.30 ± 3.75), and buffy coat (14.06 ± 4.24), (C) Within-person PC-SkinBlood epigenetic age acceleration correlations across tissues. Thick black horizontal bars on violin plots indicate the tissue-stratified median clock value and colored boxes indicate interquartile ranges. * indicate correlations with $p<0.05$.

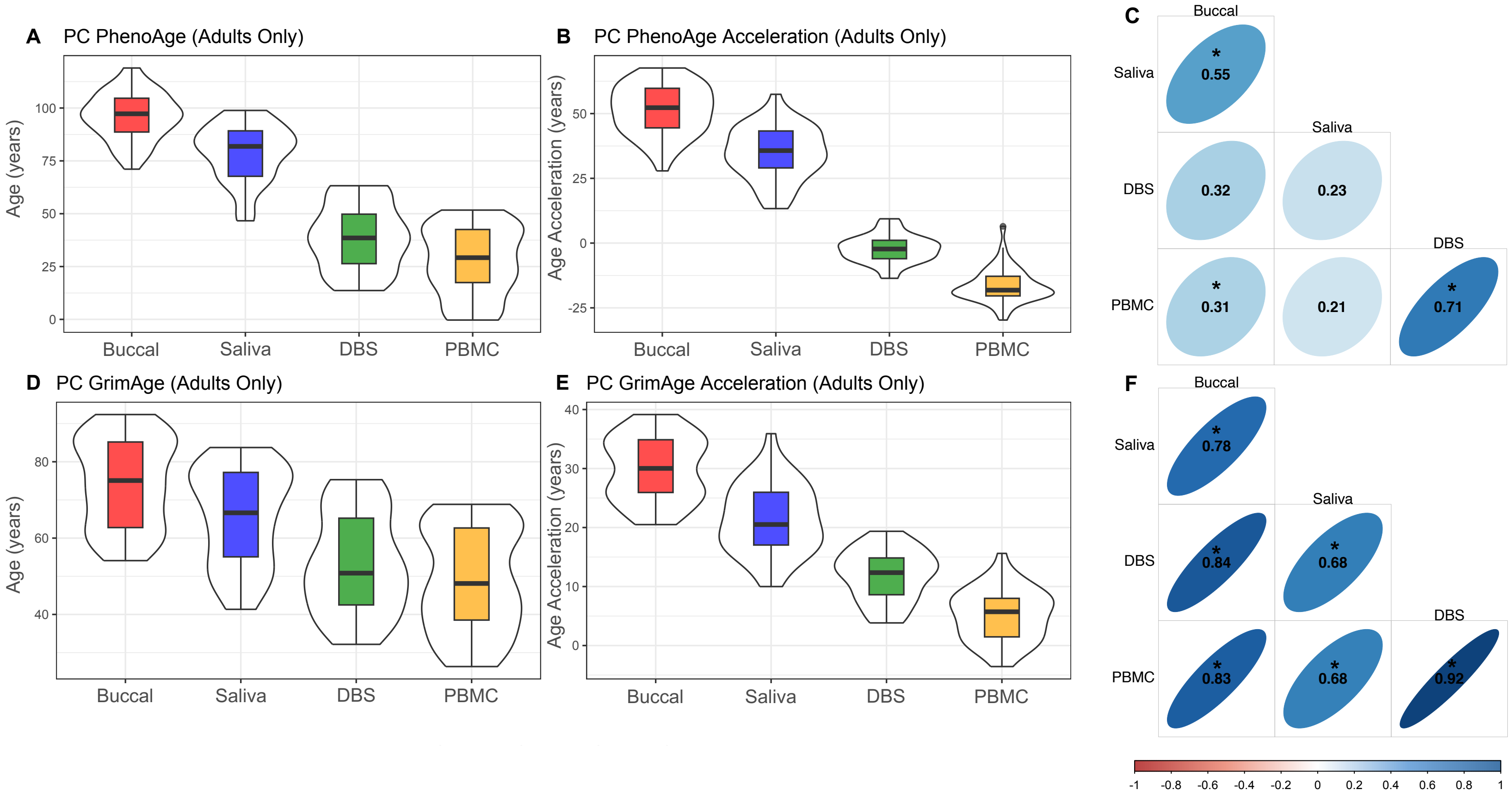

Figure S17 – Second Generation Principal Component Epigenetic Clock Distributions and Correlations (Adults Only). (A) PC-PhenoAge epigenetic age estimates for buccal (96.11 ± 11.21), saliva (78.91 ± 13.46), DBS (39.02 ± 15.44), and PBMCs (27.33 ± 16.23), (B) PC-PhenoAge epigenetic age acceleration estimates for buccal (51.49 ± 9.76), saliva (34.65 ± 10.27), DBS (-2.23 ± 5.37), and PBMCs (-16.42 ± 6.55), (C) Within-person PC-PhenoAge epigenetic age acceleration correlations across tissues, (D) PC-GrimAge epigenetic age estimates for buccal (74.65 ± 11.66), saliva (65.68 ± 12.77), DBS (53.23 ± 13.06), and PBMCs (48.89 ± 13.55), (E) PC-GrimAge epigenetic age acceleration estimates for buccal (30.03 ± 5.40), saliva (21.42 ± 5.89), DBS (11.98 ± 4.26), and PBMCs (5.15 ± 4.36) and (F) Within-person PC-GrimAge epigenetic age acceleration correlations across tissues. Thick black

horizontal bars on violin plots indicate the tissue-stratified median clock value and colored boxes indicate interquartile ranges. * indicate correlations with p<0.05.

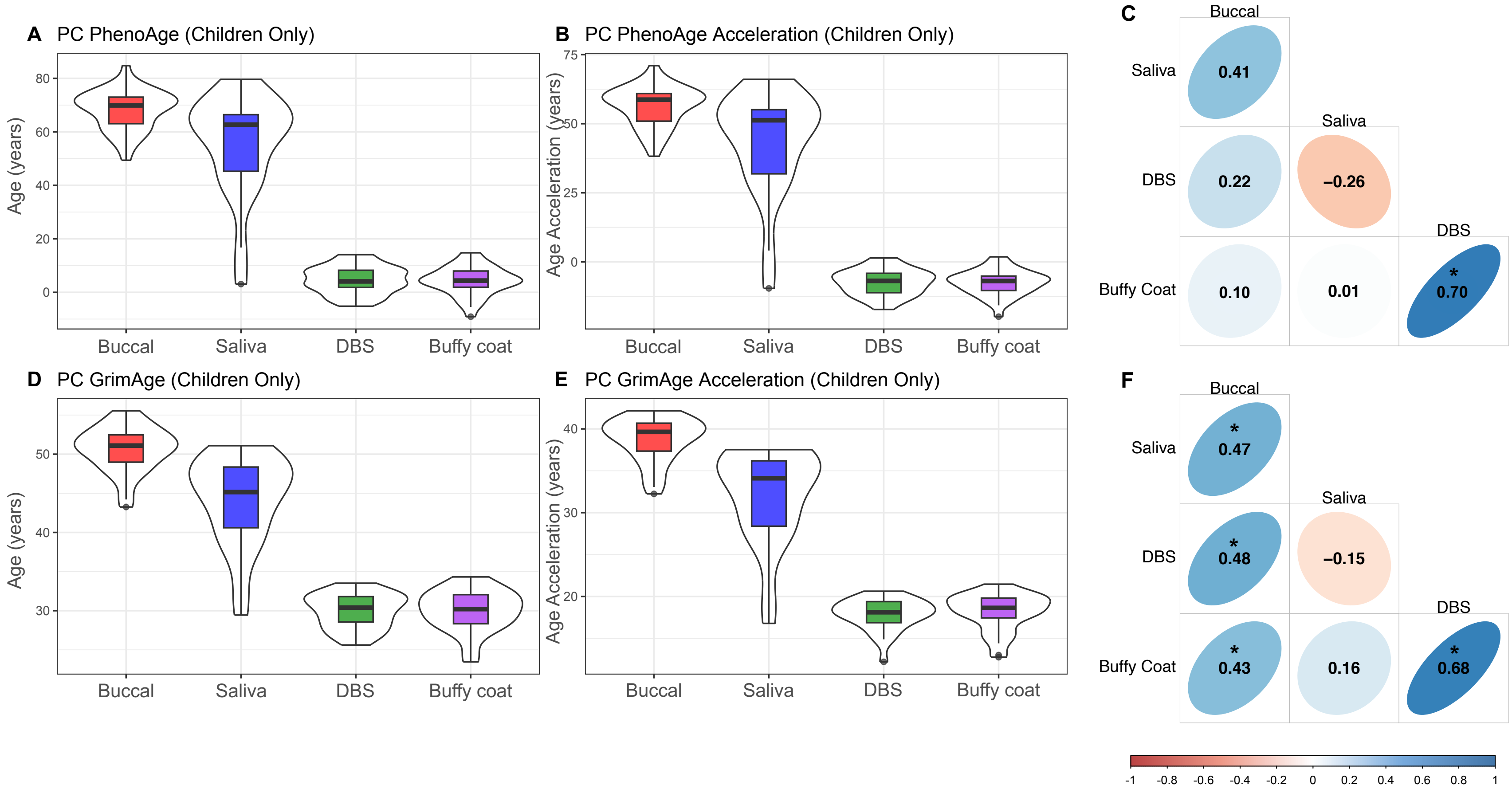

Figure S18 – Second Generation Principal Component Epigenetic Clock Distributions and Correlations (Children Only). (A) PC-PhenoAge epigenetic age estimates for buccal (68.20 ± 7.46), saliva (55.59 ± 18.84), DBS (4.65 ± 5.26), and buffy coat (4.38 ± 5.41), (B) PC-PhenoAge epigenetic age acceleration estimates for buccal (56.28 ± 7.26), saliva (43.42 ± 18.75), DBS (-7.40 ± 4.72), and buffy coat (-7.54 ± 4.68), (C) Within-person PC-PhenoAge epigenetic age acceleration correlations across tissues, (D) PC-GrimAge epigenetic age estimates for buccal (50.68 ± 2.88), saliva (44.17 ± 5.71), DBS (29.95 ± 2.21), and buffy coat (30.07 ± 2.62), (E) PC-GrimAge epigenetic age acceleration estimates for buccal (38.76 ± 2.62), saliva (31.99 ± 5.61), DBS (17.91 ± 1.86), and buffy

coat (18.16 ± 2.20) and (F) Within-person PC-GrimAge epigenetic age acceleration correlations across tissues. Thick black horizontal bars on violin plots indicate the tissue-stratified median clock value and colored boxes indicate interquartile ranges. * indicate correlations with $p<0.05$.

S2.8. Sensitivity Analyses – Epigenetic Age Accelerations Correlations Controlling for Batch and Cell Composition Effects

The following figures are correlations of epigenetic age accelerations across within-person and between-tissue that are controlled for batch effects only (**Figure S19**) and cell composition effects only (**Figure S20**) These parallel **Figure 5** from the main text.

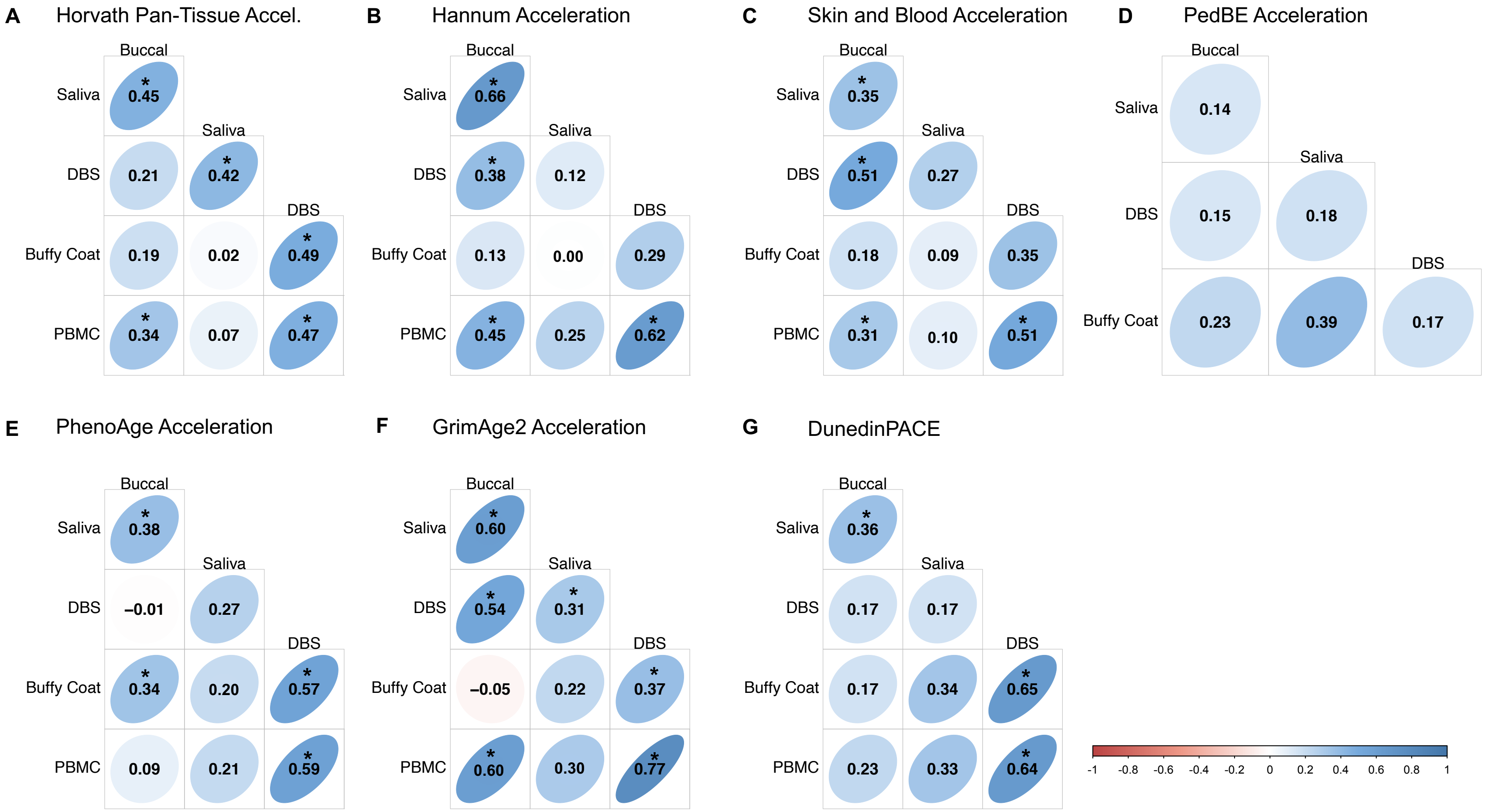

Figure S19 – Within-Person Epigenetic Age Acceleration Correlations Across Tissues Controlling for only DNAm Batch. (A) Horvath-pan tissue, (B) Hannum, (C) Skin and Blood, (D) PedBE (children only), (E) PhenoAge, (F) GrimAge2, and (G)

DunedinPACE. For all clocks, within-person correlations were residualized for DNA methylation batch. * indicate correlations with $p<0.05$.

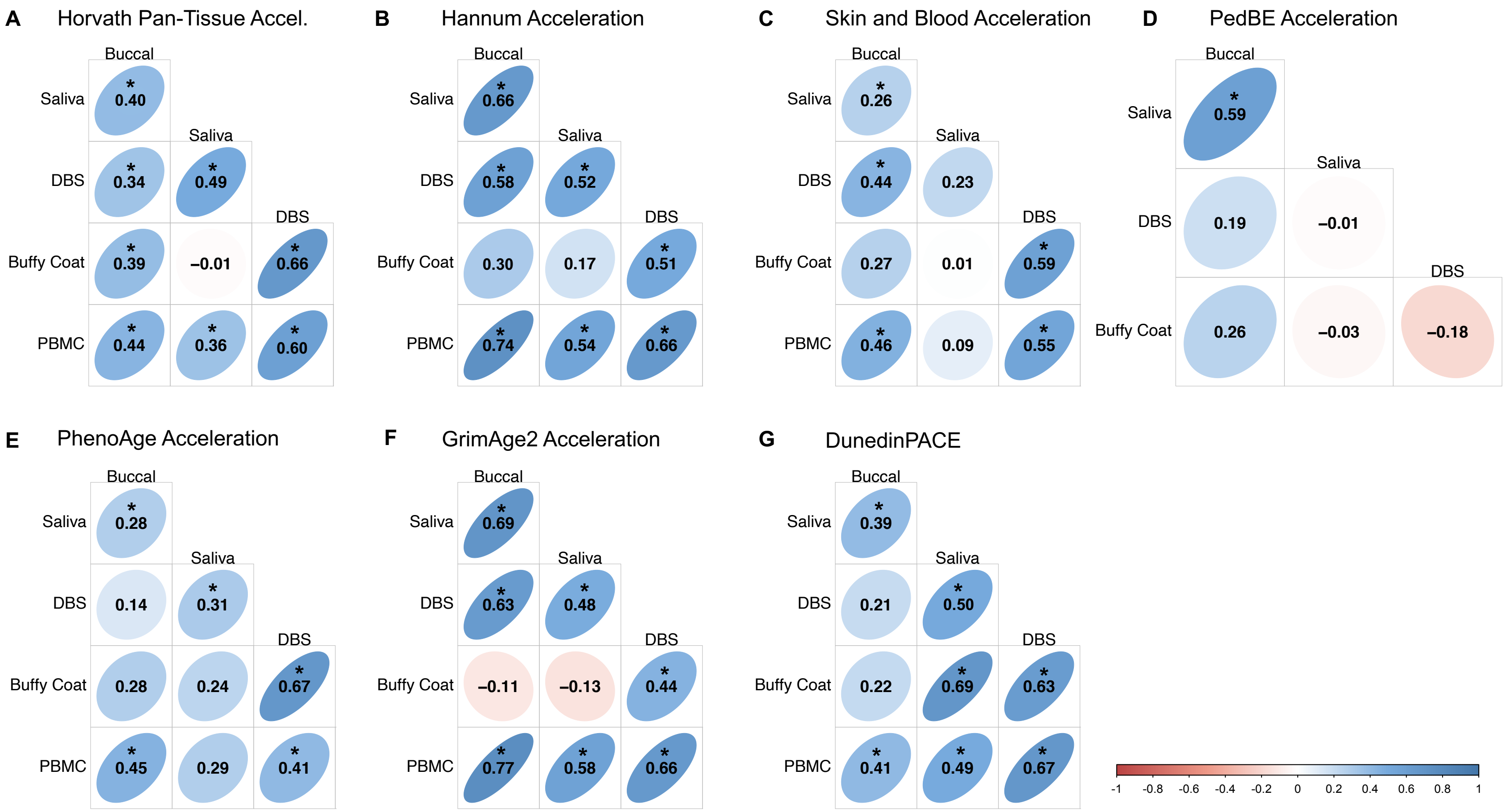

Figure S20 – Within-Person Epigenetic Age Acceleration Correlations Across Tissues Controlling for only Tissue Cellular Composition. (A) Horvath-pan tissue, (B) Hannum, (C) Skin and Blood, (D) PedBE (children only), (E) PhenoAge, (F) GrimAge2, and (G) DunedinPACE. For all clocks, within-person correlations were residualized for tissue cellular composition. * indicate correlations with $p<0.05$.
